# Analysis of *Microcystis aeruginosa* physiology by spectral flow cytometry: Impact of chemical and light exposure

**DOI:** 10.1371/journal.pwat.0000177

**Published:** 2023-10-27

**Authors:** Emma T. Brentjens, Elizabeth A. K. Beall, Robert M. Zucker

**Affiliations:** 1 Oak Ridge Institute for Science and Education Research Participation Program hosted by U.S. Environmental Protection Agency, Oak Ridge, TN, United States of America; 2 Public Health and Integrated Toxicology Division, Center for Public Health and Environmental Assessment, U.S. Environmental Protection Agency, Office of Research and Development, RTP, NC, United States of America

## Abstract

*M*. *aeruginosa* fluorescent changes were observed using a Cytek Aurora spectral flow cytometer that contains 5 lasers and 64 narrow band detectors located between 365 and 829 nm. Cyanobacteria were treated with different concentrations of H_2_O_2_ and then monitored after exposure between 1 and 8 days. The red fluorescence emission derived from the excitation of cyanobacteria with a yellow green laser (550 nm) was measured in the 652–669 nm detector while green fluorescence from excitation with a violet laser (405 nm) was measured in the 532–550 nm detector. The changes in these parameters were measured after the addition of H_2_O_2_. There was an initial increase in red fluorescence intensity at 24 hours. This was followed by a daily decrease in red fluorescence intensity. In contrast, green fluorescence increased at 24 hours and remained higher than the control for the duration of the 8-day study. A similar fluorescence intensity effect as H_2_O_2_ on *M*. *aeruginosa* fluorescence emissions was observed after exposure to acetylacetone, diuron (DCMU), peracetic acid, and tryptoline. Minimal growth was also observed in H_2_O_2_ treated cyanobacteria during exposure of H_2_O_2_ for 24 days. In another experiment, H_2_O_2_-treated cyanobacteria were exposed to high-intensity blue (14 mW) and UV (1 mW) lights to assess the effects of light stress on fluorescence emissions. The combination of blue and UV light with H_2_O_2_ had a synergistic effect on *M*. *aeruginosa* that induced greater fluorescent differences between control and treated samples than exposure to either stimulus individually. These experiments suggest that the early increase in red and green fluorescence may be due to an inhibition in the ability of photosynthesis to process photons. Further research into the mechanisms driving these increases in fluorescence is necessary.

## Introduction

Cyanobacteria, which are photosynthetic prokaryotes, proliferate rapidly to form harmful algal blooms (HABs) with detrimental effects on environmental conditions and drinking water quality. Cyanobacterial blooms constitute a public health threat due to the cyanotoxins they produce, which can cause acute and chronic illness in humans and even death in domestic animals [1–3]. Therefore, detecting and reducing the presence of cyanobacteria in water systems is critical to human and ecosystem safety.

Light absorbed by cyanobacteria may be used for photosynthesis, dissipated as heat, or emitted as fluorescence from pigments related to photosynthetic processes [4]. Phycobilisomes (PBSs), the light harvesting antennae of cyanobacteria, contain pigments that mainly excite with green and orange light and emit peak red (660- nm) fluorescence [5, 6]. Chlorophyll also emits red (685–695 nm) fluorescence predominantly when excited with blue light [460 nm; 7–9]. Changes in fluorescence emissions from these pigments may provide information regarding photosynthetic and physiological changes in cyanobacteria.

Cyanobacteria PBSs contain three main pigments: phycocyanin (PC) and allophycocyanin (APC), which are found in all cyanobacteria, and occasionally phycoerythrin (PE), which is mainly present in marine cyanobacteria [5]. PE in cyanobacteria absorbs blue and green light (absorption max. of ~565 nm), emits green light (~580 nm), and transfers energy to PC [10]. PC emits red fluorescence and transfers energy to APC [~640 nm; 11]. APC predominantly excites with red light and emits red light [~660 nm; 11, 12]. Energy is then transferred from APC to the reaction center in photosystem II [PSII; 11], the first stage of the light dependent reactions in photosynthesis [13]. Energy transfer from APC to chlorophyll a in PSII results in fluorescence emissions at 695 nm [8]. These photons are used to produce energy, generate oxygen, and drive the Calvin cycle. There is a large Stokes shift of around 100–200 nm during this process depending on the excitation wavelength. This transfer of photons from the visible range to the far-red range by phycobilisomes is essential for photosynthesis to work in cyanobacteria.

Chemicals and intense light have been proposed to affect the functioning of cyanobacteria. These treatments seem to interfere with D1 protein synthesis, the transfer of light from phycobilisomes to PSII and/or the transfer of electrons from PSII to PSI [14–19]. Inhibitions to photosynthesis in cyanobacteria are likely to increase fluorescence emissions from pigments located prior to the spectra where they are utilized for energy production and glucose generation.

Hydrogen peroxide (H_2_O_2_) is one chemical commonly added to water systems to reduce cyanobacteria growth. H_2_O_2_ is a reactive oxygen species (ROS), making it an effective chemical for targeting cyanobacteria, which are sensitive to oxidative stress [20].

H_2_O_2_ damages cyanobacteria cell structure, causing increased permeability of the cell membrane and destruction of the thylakoid membrane [17, 21]. This occurs when H_2_O_2_ forms hydroxyl radicals, which can damage membranes, proteins, and DNA [22, 23]. H_2_O_2_ is also an oxidizing agent that disrupts electron transport, resulting in the inhibition of photosynthesis [16, 24].

H_2_O_2_ also reduces photosynthetic yield and electron transport rates of PSI and PSII, inhibiting cyanobacteria growth [17, 25]. Previous research suggests that H_2_O_2_ does not damage PSII proteins directly but targets the D1 protein located in the PSII reaction center, which regulates PSII repair [17]. A damaged PSII reaction center cannot absorb light as efficiently, which affects fluorescence [17]. Thus, analyzing fluorescence can help researchers better understand how H_2_O_2_ impacts cyanobacteria physiology.

High intensity light can also disrupt photosynthesis. Multiple studies have shown that excessive light exposure results in increased fluorescence emissions from cyanobacteria [6, 26, 27]. The combination of light and chemical exposure results in increased toxicity to cyanobacteria, especially at low wavelengths [17, 18, 20, 22, 28]. For example, UV radiation is known to be harmful to a variety of organisms and ecosystems [29]. Due to the increased efficacy of pairing light with H_2_O_2_ treatments, multiple researchers have suggested that implementing H_2_O_2_ in natural water systems on sunny days may enhance the treatment [30, 31].

The goal of this study is to examine the impact of H_2_O_2_ and light stress on *M*. *aeruginosa* fluorescence using flow cytometry (FCM). We ask the following questions: 1) how do different doses of H_2_O_2_ affect *M*. *aeruginosa* fluorescence and morphology over time? 2) how does H_2_O_2_ compare to the effects of other photosynthesis inhibitory chemicals, and 3) how does *M*. *aeruginosa* fluorescence and morphology change with the addition of light exposure to H_2_O_2_ treatments? We hypothesize that the effect of H_2_O_2_ on *M*. *aeruginosa* cells results in an increased green fluorescence and decreased red fluorescence intensity in a dose- and time-dependent manner. Exposing H_2_O_2_-treated cells to bright UV-A or blue light will increase damage to cells based on wavelength, duration of exposure, and light intensity. We expect that autofluorescence data from this flow cytometry experiment may provide an indicator of stress and perturbation in *M*. *aeruginosa*.

## Methods

### Cell culture

Lab-grown cultures of *Microcystis aeruginosa*, a species commonly found in cyanobacterial blooms, were used to test the effects of H_2_O_2_ on cyanobacteria fluorescence. *M*. *aeruginosa* are round cells ranging 4–5 μm in size. This cyanobacteria species produces multiple toxins including microcystin [2]. Cells were grown in BG-11 media in 15 mL polystyrene conical tubes under a 300–350 LUX grow light (T5 Grow Light Bulbs 2 ft, 24W 6500K).

### Flow cytometry

A Cytek Aurora flow cytometer was used to obtain fluorescent data from *M*. *aeruginosa*. The system contains five lasers (355 nm UV, 405 nm violet, 488 nm blue, 561 nm yellow-green, and 640 nm red) and 64 detectors. The order, wavelength, and power of the five lasers are as follows: yellow-green (561 nm, 50 mW), violet (405 nm, 100 mW), blue (488 nm, 50 mW), red (638 nm, 80 mW), and UV (355 nm, 20 mW). The laser excitation of the cells is separated by 20 μs. The emission spectrum from each laser was collected with 8–16 detectors, each consisting of 17–25 nm in size (Cytek Biosciences Inc., Fremont, CA). The machine also measures forward scatter (FSC) with the blue laser and measures two side scatter (SSC) channels with the blue and violet lasers. FSC refers to the scatter of light around the cell and is often used as a proxy measurement for cell size. SSC is a measure of cell granularity, determined by the reflection of light off structures inside the cell [32].

Twenty-thousand cells for each sample were counted at a slow flow rate (10–20 μL/min) in a defined region of interest (ROI) in a FSC and SSC-violet cytogram. Counts were recorded within the ROI. A dual FSC and SSC threshold was used to remove the noise in the system. The flow cytometer underwent a quality assurance procedure with Spectro Flo 2000 series (lot 2003) 3 μm QC beads to check for acceptable CV values daily prior to use. The CVs of the beads measured in the majority of the 64 detectors were usually around 2–3%.

### H_2_O_2_ and chemical treatments

Five mL of a growing *M*. *aeruginosa* culture was diluted around 3x (to 16 mL) with BG-11 growth media (Sigma, St. Louis, MO). Ten doses of 3% commercial H_2_O_2_ were added to 1 mL *M*. *aeruginosa* samples at the following concentrations: 3000, 1500, 750, 300, 150, 75, 30, 15, 7.5, and 3 μg/mL. Two 1 mL control samples with no chemicals added were also included.

Samples were measured on the flow cytometer at 24 hours, 48 hours, 72 hours, and 8 days to observe how *M*. *aeruginosa* cell fluorescence and morphology changed over time in response to different H_2_O_2_ concentrations. 200 uL of each sample was measured between day 1 and day 3. At day 8, 200 uL of DI water was added to reduce the cell count to less than 1,500 per second, which also decreased the abort rate.

To determine the growth of samples treated with different doses (75–750 μg/mL) of H_2_O_2_, cells were counted with the flow cytometer at 0, 5, 8, 17 and 24 days after the addition of H_2_O_2_. The growth curves of control and treated cells in addition to the spectral charts and cytograms are displayed in the results.

Acetylacetone (product number: P7754), diuron (DCMU, product number: D2425), peracetic acid (product number: 269339), and tryptoline (product number: 300764) were obtained from Sigma-Aldrich (St. Louis, MO) added to *M*. *aeruginosa* samples to compare their effect on fluorescence to that of H_2_O_2_. The chemicals were added to 1mL *M*. *aeruginosa* samples at the following concentrations: 740, 74, 7.4 μg/mL acetylacetone (diluted to 2.97% in DI water); 0.5, 0.2, 0.1 μg/mL DCMU (diluted to 1% in ethanol); 800, 80, 8, 0.8 peracetic acid (dilute to 3.2% in DI water); and 75, 7.5, 0.75 μg/mL tryptoline (diluted to 1% in DMSO). Samples were measured on the flow cytometer at 48 and 72 hours. The different chemical treatments added to *M*. *aeruginosa* are outlined in Table 1.

### Light stress

To assess how H_2_O_2_ and light exposure interact to affect *M*. *aeruginosa* fluorescence, control and H_2_O_2_-treated *M*. *aeruginosa* cells were placed under blue and green NIGHTSEA lights (Electron Microscopy Sciences, Hatfield, PA) and 1-foot-long UV LED lamps. The cells were exposed to blue (440–460 nm) light at intensities of 14 and 7 mW, green (510–540 nm) light at intensities of 15 and 6 mW, and UV-A (395 nm) light at 1 and 0.5 mW. Different light intensities were achieved by placing the sample tubes at different distances from the light source. Light intensity was measured using a Laser Mate-Q detector (Coherent, Santa Clara, California) with either a visible or UV probe dependent on the wavelength measured. Each light treatment included control *M*. *aeruginosa* cells and H_2_O_2_-treated samples at concentrations of 750, 75, and 7.5 μg/mL. Cell fluorescence was measured on the flow cytometer after an initial 2.5 hours of continuous light exposure, an overnight recovery period under a white grow light at 350 LUX, and a second 2.5-hour light exposure, and then at 24 hours, 48 hours, 72 hours, and 8 days after initial light and H_2_O_2_ exposure. When the cells were not exposed to green, blue, or UV light, they were incubated under a standard grow light with a 12-hour light-dark cycle.

### Data analysis

Data was acquired in the Cytek Aurora flow cytometer using the Spectro Flo operating system software. The data was exported into FCS 3.0 files to enable the utilization of FCS Express (version 7.16, De Novo Software, Pasadena, CA) software that can be used to analyze the data. The samples were initially gated using a region defined by forward scatter area (blue laser) and side scatter area (violet laser). These cells from the scatter gate were then observed in a cytogram defined by the fluorescence channels YG4 (652–669 nm) on the y-axis and V7 (533–550 nm) on the x-axis to study the population of cyanobacteria. Mean light scatter and mean fluorescent data were exported into Microsoft Excel for analysis.

## Results

**Fig 1.**
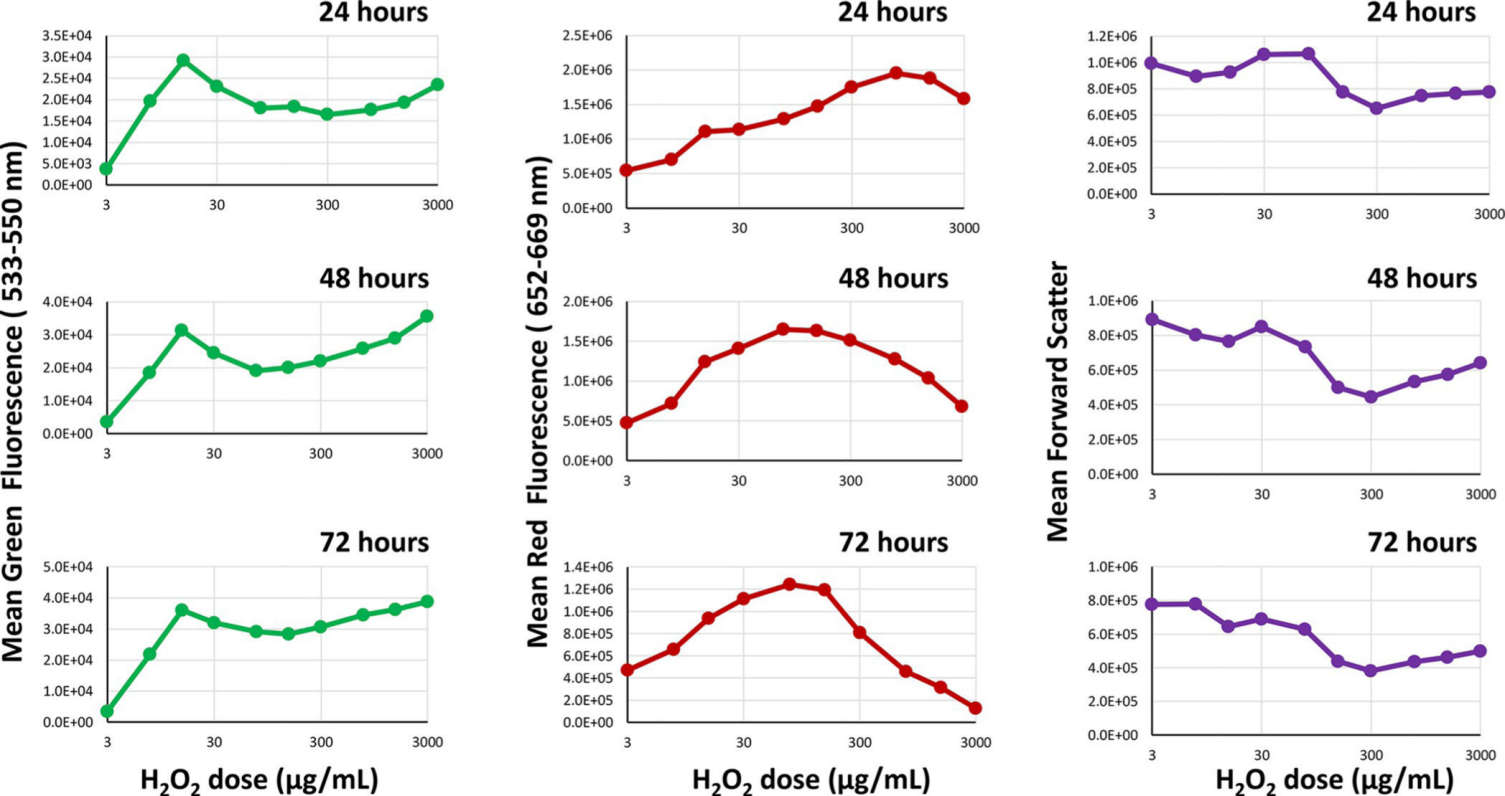
Dose dependent changes in fluorescence and forward scatter from cyanobacteria treated with H_2_O_2_. Fig 1 shows the dose dependent change in red fluorescence in one detector (652–669 nm)from a yellow green laser and green fluorescence in one detector (533–550 nm) from a violet laser for doses between 3 and 3000 μg/mL during a 3-day period (24–72 hours). There is an increase in green and red fluorescence at 24 hours. At higher doses there is an increase in green fluorescence and decrease in red fluorescence and forward light scatter relative to control cells.

The addition of H_2_O_2_ to cyanobacteria results in dose dependent changes of fluorescence and scatter between 24 and 72 hours. For these studies photosynthesis emission ranges of 600 to 829 nm were designated as “red fluorescence” and the spectral ranges between 450 and 580 nm were designated as “green fluorescence.” There was an initial increase in both red and green fluorescence at 24 hours. At higher doses the red fluorescence decreases after 48 and 72 hours while the green fluorescence does not decrease (Fig 1). The forward light scatter generally decreases at most concentrations compared to control cells.

**Fig 2.**
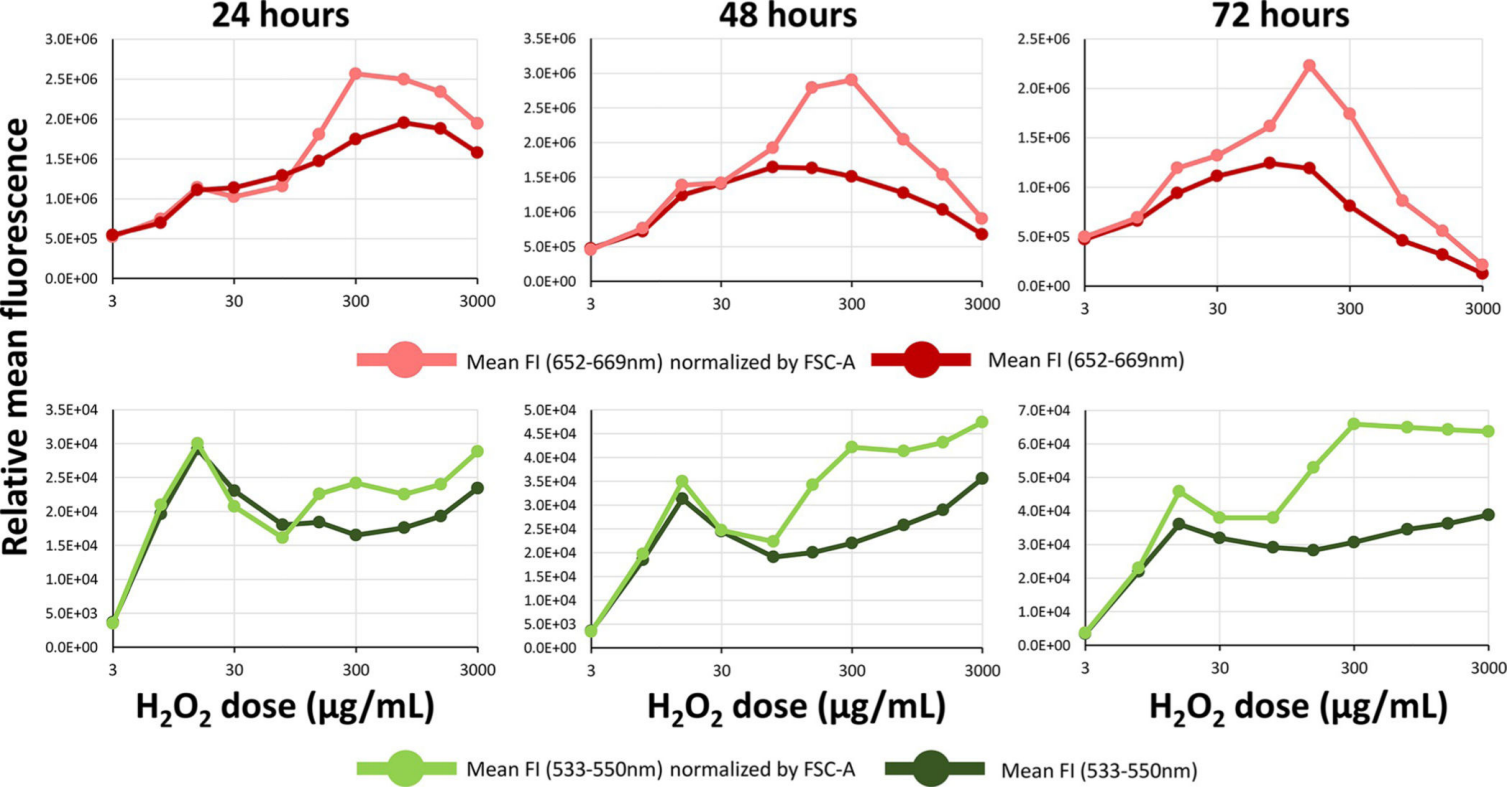
Dose dependent changes in fluorescence from cyanobacteria treated with H_2_O_2_ after normalization with forward scatter. By dividing the green fluorescence (lower panels) and red fluorescence (upper panels) by the relative forward light scatter parameter shown in Fig 1,the graphs show that the resultant fluorescence actually increased with almost all doses with the highest values being at around 3000 μg/mL for red fluorescence. Green points represent values derived from the violet laser using the green detector (533–550 nm) and red points represent intensity values derived from the yellow-green laser in the red detector range (652–669 nm).

The decrease in forward light scatter may influence the detection of green and red fluorescence from the cyanobacteria. To normalize for the effect that size may have on fluorescence intensity emission after incubation of cyanobacteria with H_2_O_2_, the fluorescence measurements were divided by the relative change in forward scatter. Even after this normalization procedure, an increase in fluorescence after treatment with H_2_O_2_ was apparent (Fig 2).

**Fig 3.**
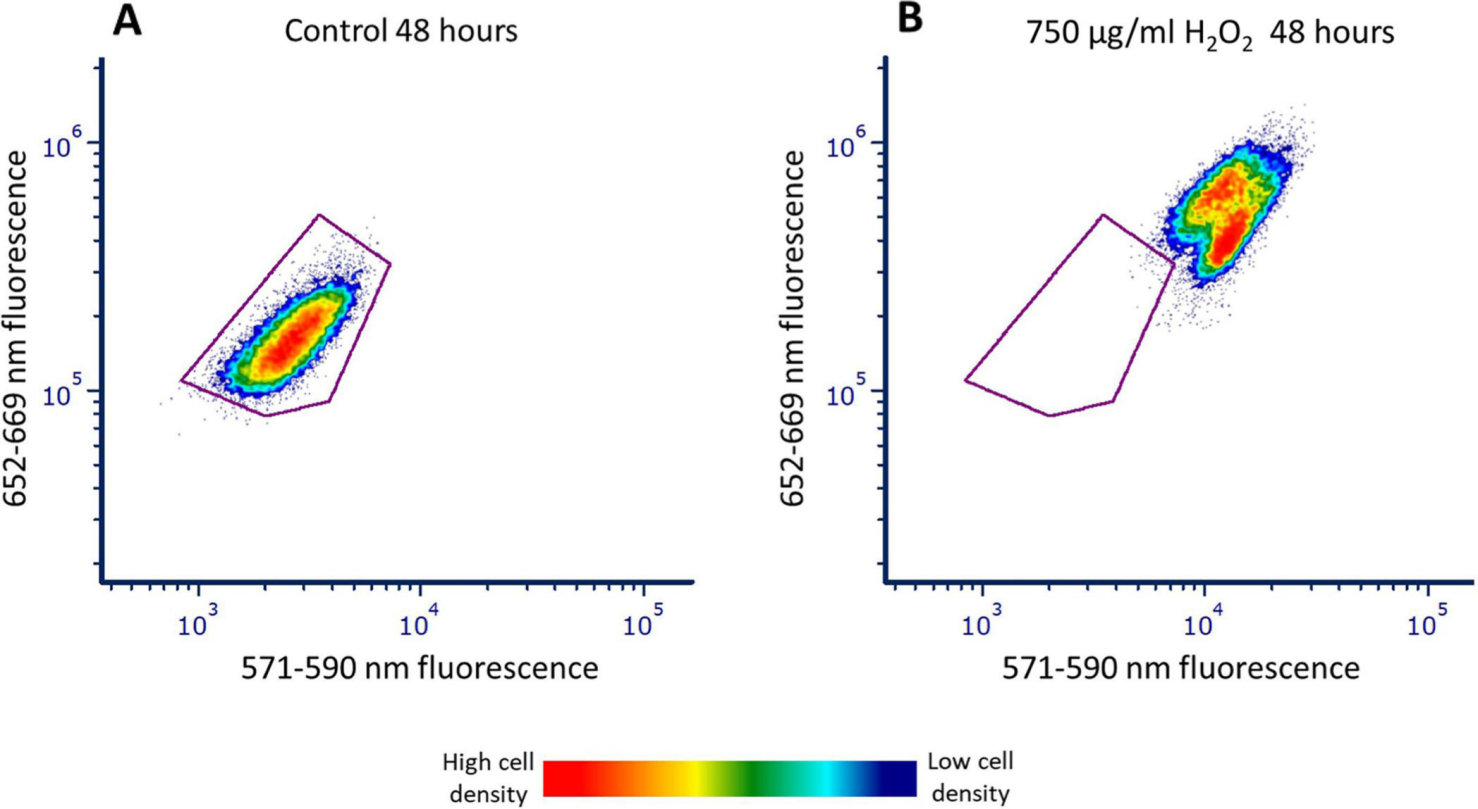
The detection of red (652–669 nm) and green (533–550 nm) fluorescence from yellow-green and violet laser excitation in control and H_2_O_2_-treated *M*. *aeruginosa* after 48 hours. Density cytograms show red fluorescence from 561 nm laser excitation and green fluorescence from the 405 nm laser excitation. There is an increase in both red and green fluorescence in the 750 μg/mL H_2_O_2_ sample (B) relative to the control (A) after 48 hours of exposure. The red color in the figure represents higher density of cells while dark blue represents lower density of cells. The purple outline indicates the control region (based on the population in A). B shows the movement of cells out of the control region (A) after incubation with H_2_O_2_ for 48 hours.

The flow cytometer detected an increase in both red (615–829 nm) and green fluorescence (458–588 nm) in *M*. *aeruginosa* after 48 hours of exposure to 750 μg/mL H_2_O_2_ relative to the control as shown in the cytogram (Fig 3). The data for 48-hour control and H_2_O_2_ treated cells are displayed as a cytograms consisting of green fluorescence emitted from violet laser excitation (533–550 nm) vs red fluorescence emitted from yellow-green laser excitation (652–669 nm) (Fig 3).

**Fig 4.**
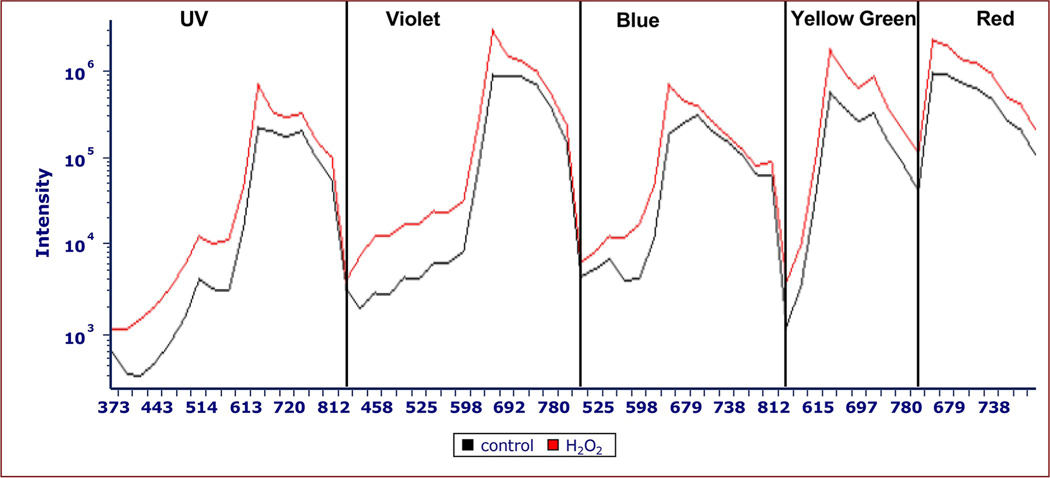
Fluorescence intensity curves of control and 750 μg/mL H_2_O_2_ *M*. *aeruginosa* samples from UV, violet, blue, yellow-green, and red laser excitation. Intensity curves show relative fluorescence intensity (y-axis) with UV (355 nm), violet (405 nm), blue (488 nm), yellow-green (561 nm), and red (640 nm) laser excitation of control (black) and 750 μg/mL H_2_O_2_ (red) cells at 48 hours of exposure. Intensity curves were generated by calculating the mean relative fluorescence intensity in each channel using FCS Express version 7.16 The x-axis shows the individual detectors designated by Cytek. The emissions occur from the following ranges: UV: 372 to 829 nm in 16 detectors; violet: 420 to 829 nm in 16 detectors; blue: 498 to 829 nm in 14 detectors; yellow-green: 567 to 829 nm in 10 detectors; and red (660 to 829 nm in 8 detectors. There are ~660 nm fluorescence peaks from the H_2_O_2_-treated sample, while the control sample does not exhibit this increase in fluorescence emissions at 660 nm. H_2_O_2_-treated cells showed an increase in the green fluorescence ranges with violet and blue laser excitation.

The increase in red and green fluorescence was displayed as Cytek fluorescence intensity spectra (Fig 4). Intensity curves were generated by calculating the mean relative fluorescence intensity in each channel using FCS Express version 7.16 The x-axis shows the individual detectors designated by Cytek. Intensity curves show relative fluorescence intensity (y-axis) with UV (355 nm), violet (405 nm), blue (488 nm), yellow-green (561 nm), and red (640 nm) laser excitation of control (black) and 750 μg/mL H_2_O_2_ (red) cells at 48 hours of exposure. The emissions occur from the following ranges: UV: 372 to 829 nm in 16 detectors; violet: 420 to 829 nm in 16 detectors; blue: 498 to 829 nm in 14 detectors; yellow-green: 567 to 829 nm in 10 detectors; and red (660 to 829 nm in 8 detectors.

**Fig 5.**
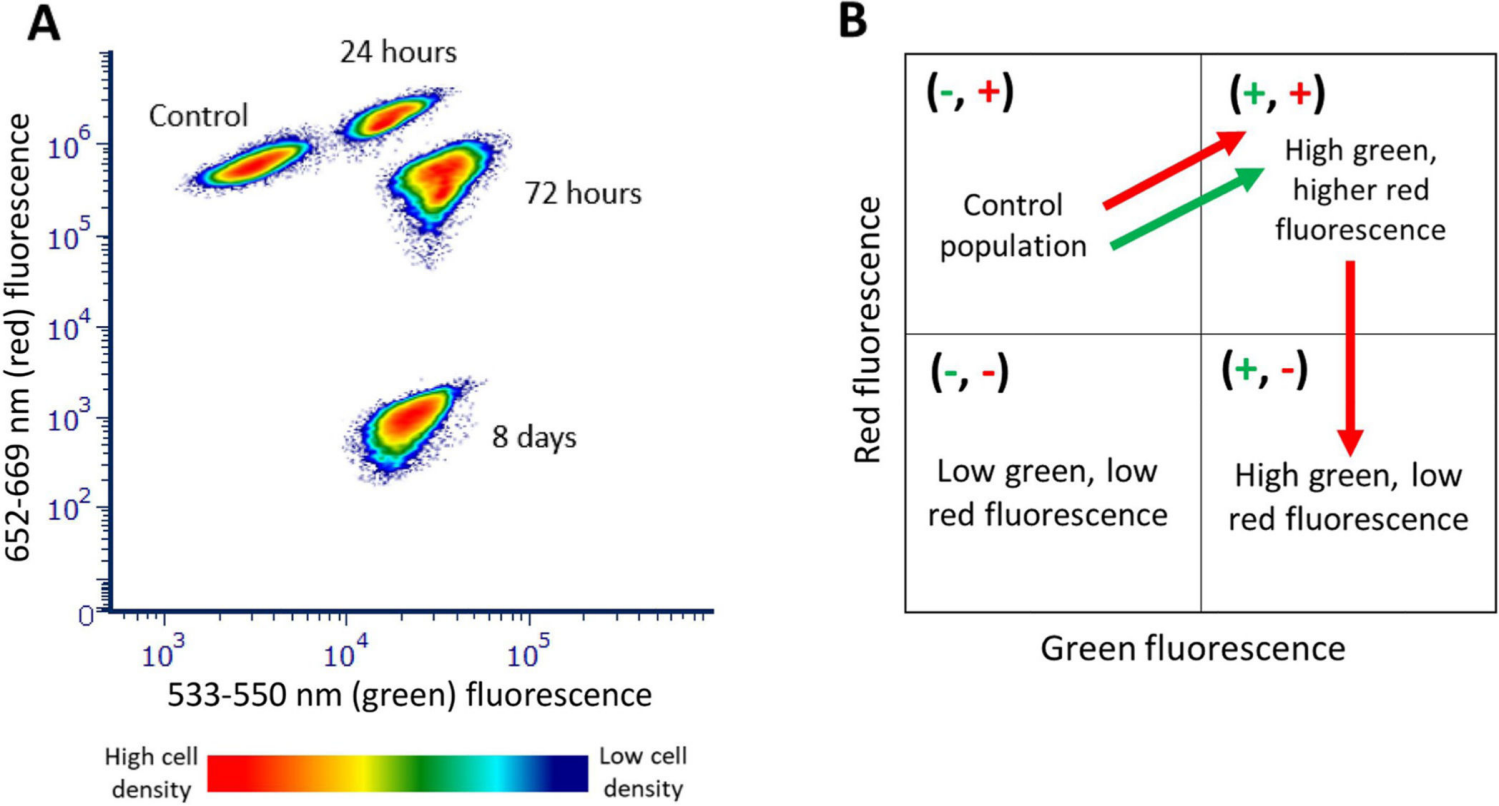
Pattern of *M*. *aeruginosa* fluorescence change with exposure to 750 μg/mL H_2_O_2_. A is a density cytogram showing red fluorescence (emission: 652–669 nm, excitation: 561 nm) on the y-axis and green fluorescence (emission: 533–550 nm, excitation: 405 nm) on the x-axis of control and 750 μg/mL H_2_O_2_ *M*. *aeruginosa* samples after 24 hours, 72 hours, and 8 days. Red represents high density of cells and dark blue represents low density. B is a diagram showing the direction of *M*. *aeruginosa* fluorescence changes. Control *M*. *aeruginosa* populations began with high red fluorescence and low green fluorescence emissions; red and green fluorescence increased 24 hours later, then red fluorescence decreased after 72 hours and 8 days.

Fig 5 demonstrates a summary of the time- and dose-dependent effect of H_2_O_2_ on *M*. *aeruginosa* fluorescence. The increase in red fluorescence was followed by a decrease after 72 hours, while the increased green fluorescence relative to the control was maintained after 8 days of H_2_O_2_ exposure (Fig 5). The addition of H_2_O_2_ caused a dose- and time-dependent change in red and green fluorescence, with red fluorescence emissions initially increasing by up to 240% relative to the control before eventually decreasing by about 1000 times as shown in Fig 5. In contrast to red fluorescence, green fluorescence increased at 24 hours by up to about 570% and remained higher than the control for the duration of the 8-day study.

The red fluorescence of most treated samples was greater than the control after 24 and 48 hours of H_2_O_2_ exposure (Table 2). On day 8, almost all treated samples had a lower red fluorescence (ratio *<* 1) than the control (Table 2). Green fluorescence emissions in most of the treated samples increased steadily in the first 72 hours of exposure. Green fluorescence began to decline in samples with H_2_O_2_ concentrations of 300 μg/mL and greater by day 8. However, these samples still exhibited a higher green fluorescence (ratio *>* 1) than the control on day 8 (Table 2).

**Fig 6.**
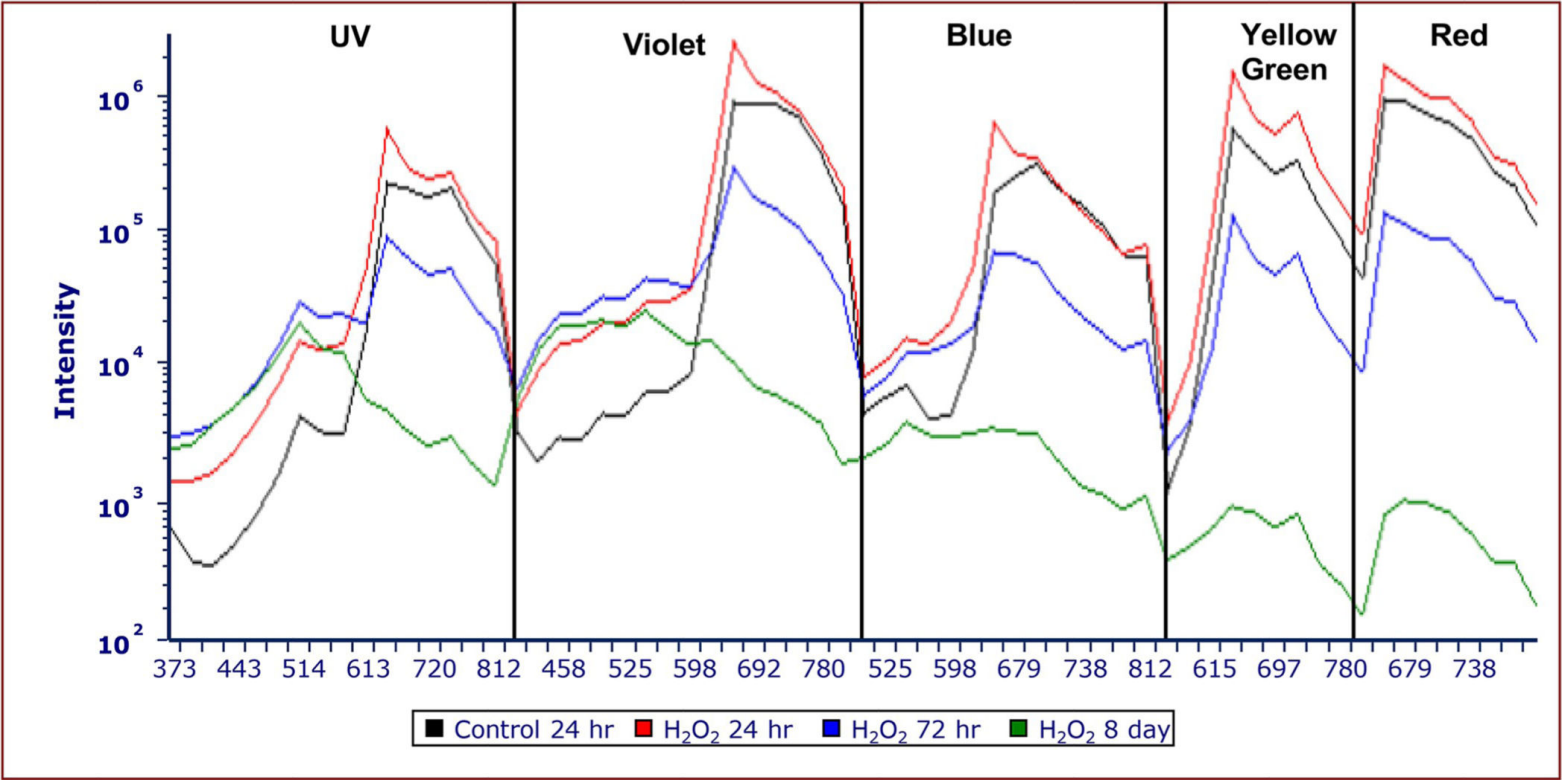
Fluorescence intensity curves of control and 750 μg/mL H_2_O_2_ *M*. *aeruginosa* samples from UV, violet, blue, yellow-green, and red laser excitation. Intensity curves show fluorescence emission intensity (y-axis) derived with five lasers: UV (355 nm), violet (405 nm), blue (488 nm), yellow-green (561 nm) and red (640 nm). The excitation of control (black, 24 hour) and 750 μg/mL H_2_O_2_ cells at 24 hours (red), 72 hours (blue), and 8 days (green). Intensity curves were generated by calculating the mean fluorescence intensity in each channel using FCS Express version 7.12. The x-axis shows the individual detectors designated by Cytek. The emissions occur from the following ranges: UV: 372 to 829 nm in 16 detectors; violet: 420 to 829 nm in 16 detectors; blue: 498 to 829 nm in 14 detectors; yellow-green: 567 to 829 nm in 10 detectors; and red: 660 to 829 nm in 8 detectors. There are ~660 nm fluorescence peaks from the H_2_O_2_-treated sample, while the control sample does not exhibit this increase in fluorescence emissions at 660 nm. H_2_O_2_-treated cells showed an increase in the green fluorescence ranges with UV, violet, and blue laser excitation at all time points.

Fig 6 shows a spectral map of the five lasers across all the detectors that correlates to the cytograms in Fig 5. At 24 hours, green and red fluorescence emissions from the H_2_O_2_-treated cells was greater than the control in all 64 diode detectors than the control. At 72 hours, the fluorescence in the red range was less than the control while the fluorescence in the green range was greater than the control cells. At 8 days, the green fluorescence from the UV, violet, and blue lasers was greater while the red fluorescence was much lower than the control. The increased fluorescence after H_2_O_2_ exposure appears more pronounced in the blue and green emission spectral ranges at all time points with the UV and violet, lasers excitation (Fig 6). The intensity curves also reveal a fluorescence peak at around 660 nm from the violet, blue, and yellow-green lasers (Fig 6). This peak is not present in control cells with violet and blue laser excitation (Fig 6). Treated and control cells exhibit peaks at 660 nm and 697 nm with yellow-green laser excitation, which are higher in treated samples after 24 and 48 hours (Fig 6).

**Fig 7.**
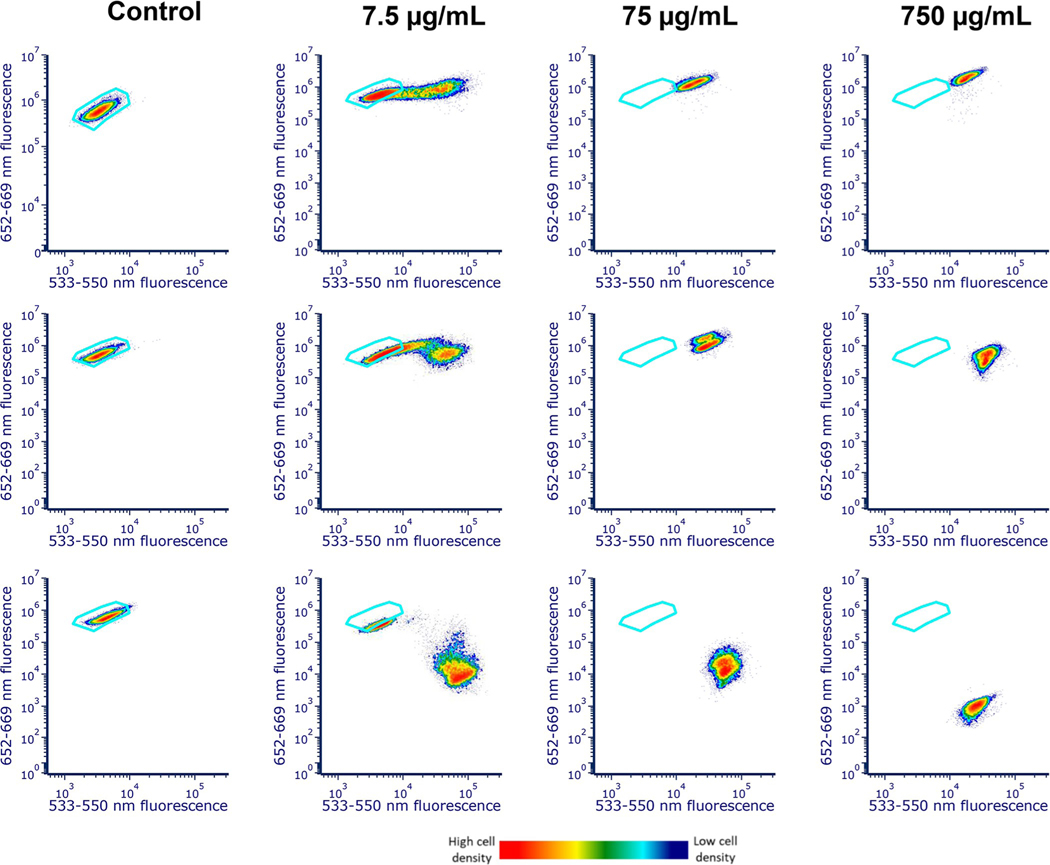
Dose- and time-dependent response of *M*. *aeruginosa* fluorescence to H_2_O_2_ exposure. The density cytograms show red fluorescence (emission: 652–669 nm, excitation: 561 nm) on the y-axis and green fluorescence (emission: 533–550 nm, excitation: 405 nm) on the x-axis of control, 750 μg/mL, 75 μg/mL, and 7.5 μg/mL H_2_O_2_ *M*. *aeruginosa* samples after 24 hours, 72 hours, and 8 days. Red represents a high density of cells and dark blue represents low density of the cells. The blue gate shown in all the cytograms represents the region containing control cells and serves as a reference for changes in fluorescence of H_2_O_2_ treated cells. The population located within the control region decreased to 0% after 24 hours for both the 750 and 75 μg/mL H_2_O_2_ samples. In contrast, the percentage of cells treated with low dose (7.5 μg/mL) H_2_O_2_ found in the control region decreased each day and remained around 30% on day 8.

Overall, red fluorescence eventually decreased with greater H_2_O_2_ exposure time while green fluorescence generally increased over the same period. Fig 7 further illustrates dose- and time-dependent effects of H_2_O_2_ on *M*. *aeruginosa* fluorescence. Over time and with higher doses of H_2_O_2_, treated populations show increased fluorescence intensity and shift farther to the right (greater green fluorescence) from the control region. In the higher concentration (750 μg/mL), 0% of the population remains in the control region after 72 hours while about 32% of the lower concentration (7.5 μg/mL) sample remains in the control region after 8 days.

*M*. *aeruginosa* cells also exhibited changes in FSC and SSC in response to H_2_O_2_ exposure. Both FSC and SSC generally decreased in relative size with increasing H_2_O_2_ concentration (S1 Table). At 48 hours, each dose used with the exception of the 30 μg/mL dose had lower FSC and SSC values compared to that of the control. FSC was also negatively associated with exposure time, decreasing over the 8-day study period in most samples (S1 Table).

The observed pattern of increased red and green fluorescence shown in the cytograms (Figs 5 and 7) was consistent (Table 3). The cyanobacteria were treated with H_2_O_2_ in three separate experiments. The green and red fluorescence of the control and two doses of H2O2 was averaged to derive the mean fluorescence and standard deviation. These experiments show an increase in green fluorescence which was maintained at 48 and 72 hours. An initial increase in red fluorescence intensity was followed by a decrease in intensity.

**Fig 8.**
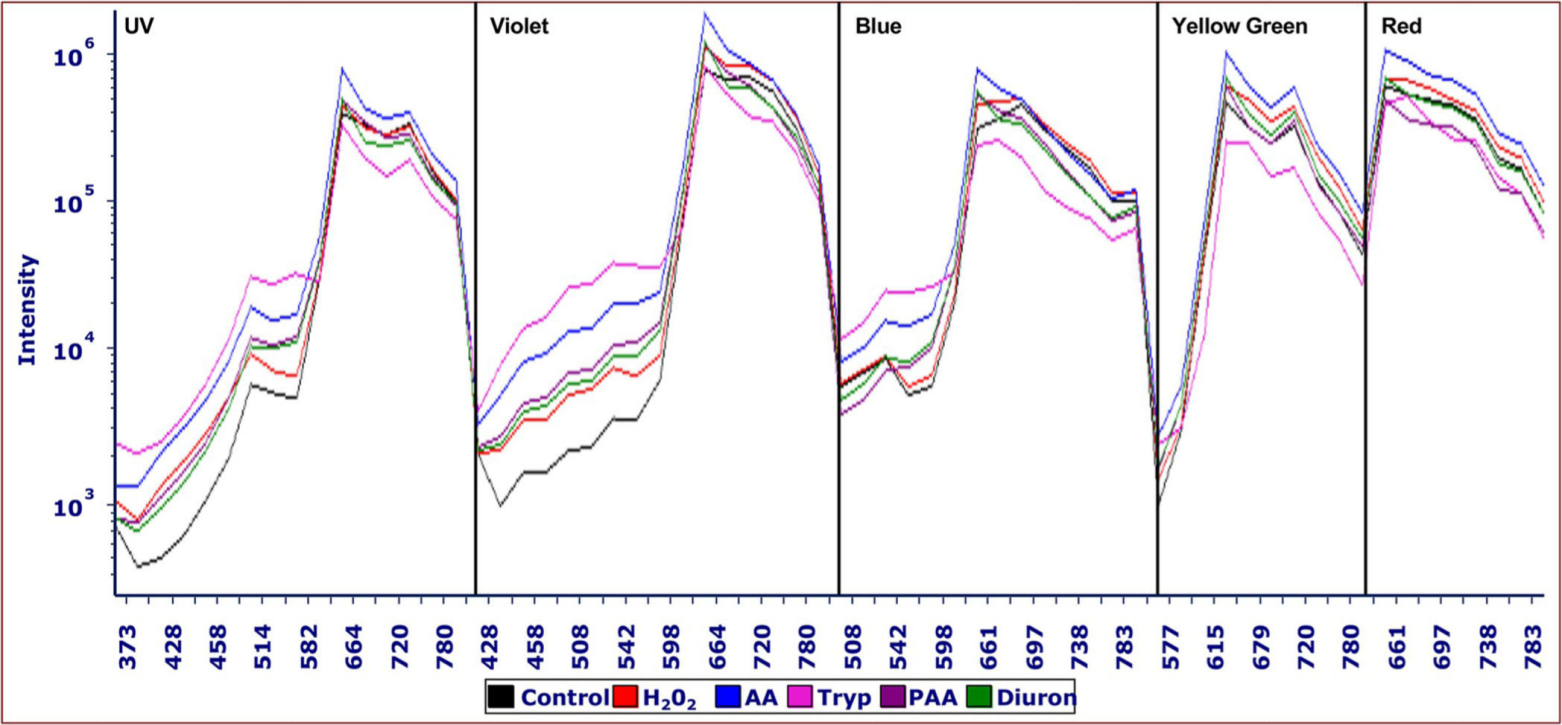
Fluorescence intensity curves of *M*. *aeruginosa* treated with PSII inhibitory chemicals from violet, blue, and yellow-green laser excitation. Intensity curves show fluorescence detected from UV (355 nm), violet (405 nm), blue (488 nm), yellow-green (561 nm), and red (640 nm) laser excitation for control (black) and 750 μg/mL H_2_O_2_-treated cells (red) after 48 hours compared to 740 μg/mL acetylacetone (blue), 200 μg/mL DCMU (green), 8 μg/mL peracetic acid (purple), and 75 μg/mL tryptoline (pink). Intensity curves were generated by calculating the mean relative fluorescence intensity in each channel. The x-axis shows the individual detectors designated by Cytek. The emissions occur from the following ranges: UV: 372 to 829 nm in 16 detectors; violet: 420 to 829 nm in 16 detectors; blue: 498 to 829 nm in 14 detectors; yellow-green: 567 to 829 nm in 10 detectors; and red: 660 to 829 nm in 8 detectors. Similar to H_2_O_2_, the acetylacetone, DCMU, and peracetic acid samples exhibited fluorescent emission peaks at around 660 nm with excitation from all five lasers, but to a lesser extent. These three compounds and H_2_O_2_ showed an increase in the green fluorescence ranges from UV, violet, and blue laser excitation. This peak at 660 nm was also observed with the violet laser in the tryptoline sample, but not with the blue or yellow-green lasers.

Fig 8 shows that *M*. *aeruginosa* fluorescence was affected by acetylacetone (AA), diuron (DCMU), peracetic acid (PAA) and tryptoline (tryp) in a similar manner as H_2_O_2_ at various doses. Each chemical caused increased fluorescence emissions in the green spectral range and all samples except tryptoline caused increased red fluorescence at 48 hours of exposure compared to control. The largest green fluorescence effects were observed with the violet laser, although the trend of increased fluorescence could be observed with the UV, blue and yellow-green lasers, as well. The H_2_O_2_, acetylacetone, DCMU, and peracetic acid all caused a similar peak at 660 nm relative to the control with all the lasers (Fig 8). It should be emphasized that only one dose of these chemicals is displayed; the point of Fig 8 was primarily to show that the effect observed with H_2_O_2_ also occurred with other chemicals that affected cyanobacteria.

**Fig 9.**
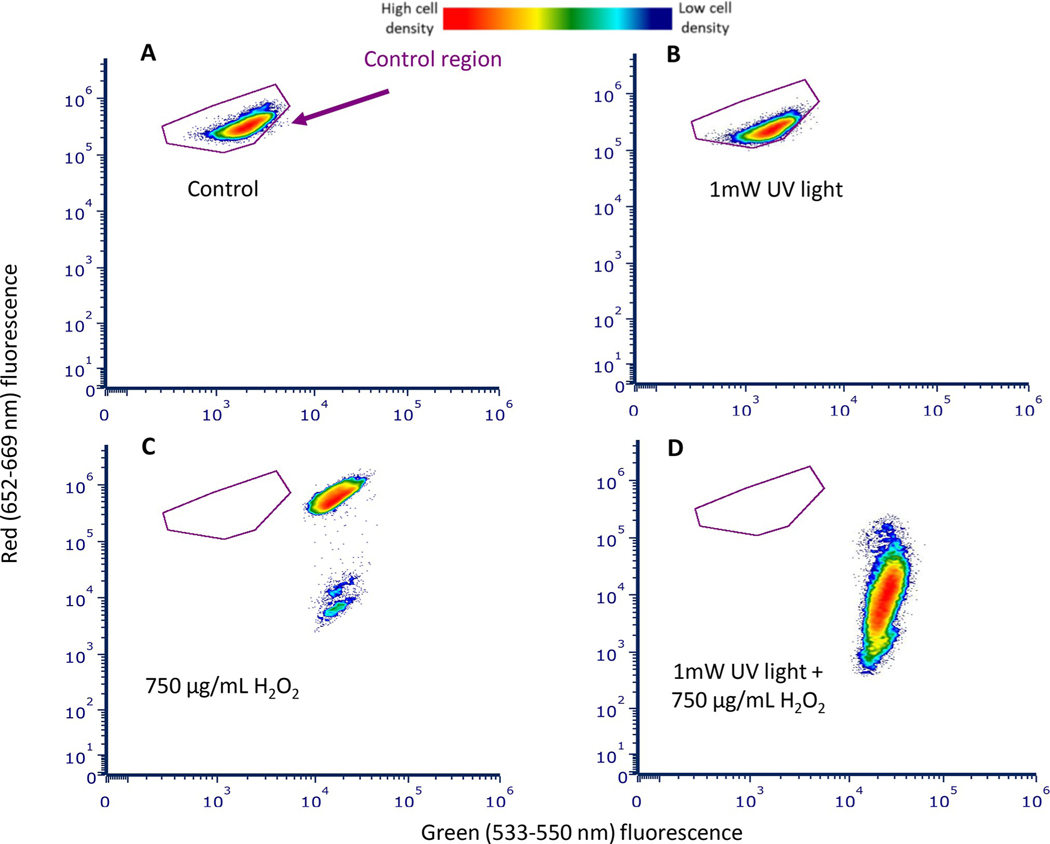
Red and green fluorescence of *M*. *aeruginosa* after exposure to different wavelength light and H_2_O_2_ at 72 hours. Density cytograms show red fluorescence (emission: 652–669 nm, excitation: 561 nm) on the y-axis and green fluorescence (emission: 533–550 nm, excitation: 405 nm) on the x-axis. In the cytograms, red represents higher density of cells and dark blue represents a lower density of cells. The purple gate represents the control region. Panel A consists of control cells (no treatment) and panel C consists of cells treated for 72 hours with 750 μg/mL H_2_O_2_. Panel B consists of cells treated with 1mw UV-A light and panel D consist of cells treated with 1 mW UV-A light and 750 μg/mL H_2_O_2_. Samples were exposed to UV-A light for 2.5 hours immediately after H_2_O_2_ exposure and for another 2.5 hours at 24 hours. After the cells were exposed to UV-A lights for 2.5 hours, they were put under grow lights with a 12-hour light-dark cycle. UV light had a synergistic effect with H_2_O_2_ on *M*. *aeruginosa* fluorescence.

Wavelengths below 460 nm had the greatest effect on H_2_O_2_-treated *M*. *aeruginosa* fluorescence. The effect of the H_2_O_2_ + light exposure treatments varied by light intensity, wavelength, exposure time, and H_2_O_2_ dose. Green light treatments did not noticeably impact *M*. *aeruginosa* fluorescence emissions while blue (440–460 nm) and UV-A (395 nm) light had a synergistic effect on changes in *M*. *aeruginosa* fluorescence when paired with H_2_O_2_ treatments (Fig 9). The effect of blue and UV-A light alone without H_2_O_2_ was not as great as the effect of H_2_O_2_ alone. However, when paired together, H_2_O_2_ and light caused greater changes in fluorescence, with green fluorescence emissions increasing and red fluorescence emissions decreasing at a faster rate. The greatest effect in these studies was observed in the 1 mW UV-A + 750 μg/mL H_2_O_2_ sample. Red fluorescence had already begun to decrease to levels below the control after the second 2.5-hour light exposure (Fig 9). At 72 hours, red fluorescence decreased to levels below the control (Fig 9).

The high light intensity treatments (14 mW blue light and 1 mW UV-A light) had a greater effect on fluorescence than the low intensity treatments (7 mW blue light and 0.5 mW UV-A light; Table 4). Additionally, like control + H_2_O_2_ cells, H_2_O_2_ in light-exposed cells had a dose- and time-dependent effect on *M*. *aeruginosa* fluorescence, with higher doses of H_2_O_2_ causing a greater increase in green fluorescence and decrease in red fluorescence over time (Table 4).

The values in Table 4 indicate the ratio of red (emission: 652–669 nm, excitation: 561 nm) and green (emission: 533–550 nm, excitation: 405 nm) fluorescence relative to the control sample (0 μg/mL H_2_O_2_, no light treatment). The effect of H_2_O_2_ and light exposure on red fluorescence was most pronounced in the 1 mW UV-A treatment, followed by the 14-mW blue treatment. The same treatments also elicited the greatest increase in green fluorescence. Green fluorescence intensity had begun to decrease after 72 hours in the 750 μg/mL H_2_O_2_ + 1 mW UV treatment and 8 days in the 750 μg/mL H_2_O_2_ + 14 mW blue light treatment, but still remained higher than green fluorescence in the control sample. Similar changes were observed at 24 and 48 hours but the magnitude of the change was not as great as that observed with the 72-hour point that showed minimal effects with only UV-A light but a synergistic effect when UV-A light was combined with H_2_O_2._

**Fig 10.**
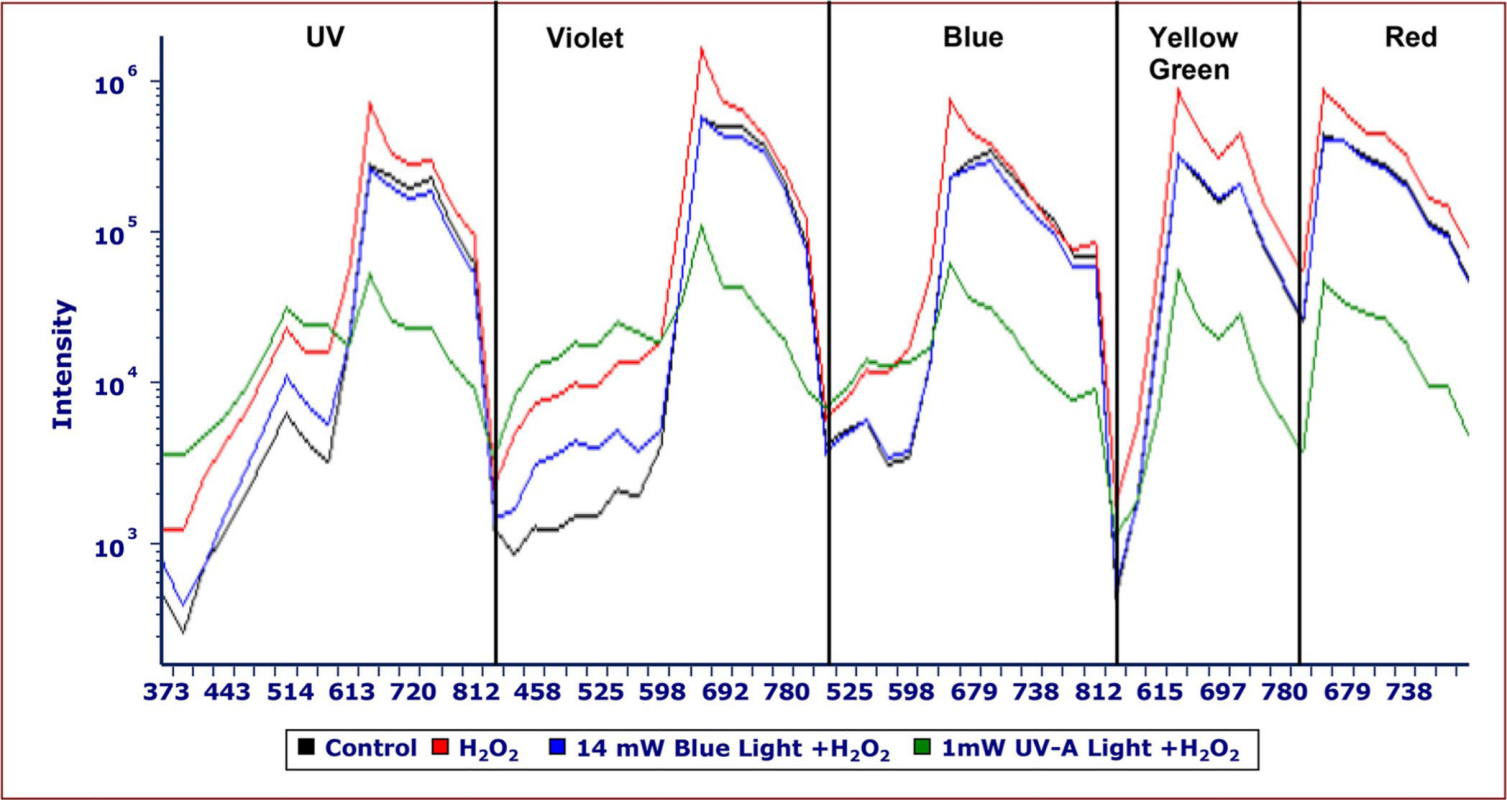
Fluorescence intensity curves of *M*. *aeruginosa* after H_2_O_2_ and light treatments from UV, violet, blue, yellow-green, red laser excitation. Intensity curves show relative emission fluorescence intensity (y-axis) derived with five lasers: UV (355 nm), violet (405 nm), blue (488 nm), yellow-green (561 nm) and red (640 nm). Cells were treated with 1) UV-A + H_2_O_2_ 2) blue light + H_2_O_2_ and 3) H_2_O_2_ and their fluorescence were compared. Cells exposed to both 14 mW blue (blue) and 1 mW UV-A light (green) showed increased fluorescence in the blue and green spectral ranges from UV, violet, and blue laser excitation compared to cells treated only with 750 μg/mL H_2_O_2_ (red). The UV-A + H_2_O_2_-treated cells had the greatest decline in red fluorescence from all five lasers. Intensity curves were generated by calculating the mean relative fluorescence intensity in each channel using FCS Express version 7.16. The x-axis shows the individual detectors designated by Cytek. The emissions occur from the following ranges: UV: 372 to 829 nm in 16 detectors; violet: 420 to 829 nm in 16 detectors; blue: 498 to 829 nm in 14 detectors; yellow-green: 567 to 829 nm in 10 detectors; and red: 660 to 829 nm in 8 detectors.

Cells were treated with 1) UV-A + H_2_O_2_ 2) blue light + H_2_O_2_ and 3) H_2_O_2_ and their fluorescence were compared. Fig 10 further demonstrates the synergistic effect of 750 μg/mL H_2_O_2_ paired with 14 mW blue or 1 mW UV-A light. The treatments with light and H_2_O_2_ showed lower red fluorescence, indicating the synergistic effect of light interacting with H_2_O_2_ (Figs 9 and 10). Each curve shows singular peaks at 660 nm with UV, violet, and blue laser excitation and peaks at 660 nm and 697 nm with yellow-green excitation.

Among the light and H_2_O_2_-treated samples, FSC and SSC also varied in a dose- and time-dependent manner. The ratio of both FSC and SSC to the control decreased over time in most samples. Additionally, the highest H_2_O_2_ concentrations (750 μg/mL) generally exhibited the lowest FSC and SSC values relative to the control at a given time point (S2 Table). One exception to this trend was the 7-mW blue light + 750 μg/mL H_2_O_2_ sample, which had considerably higher FSC and lower SSC measurements than the rest of the samples at every stage of data collection.

**Fig 11.**
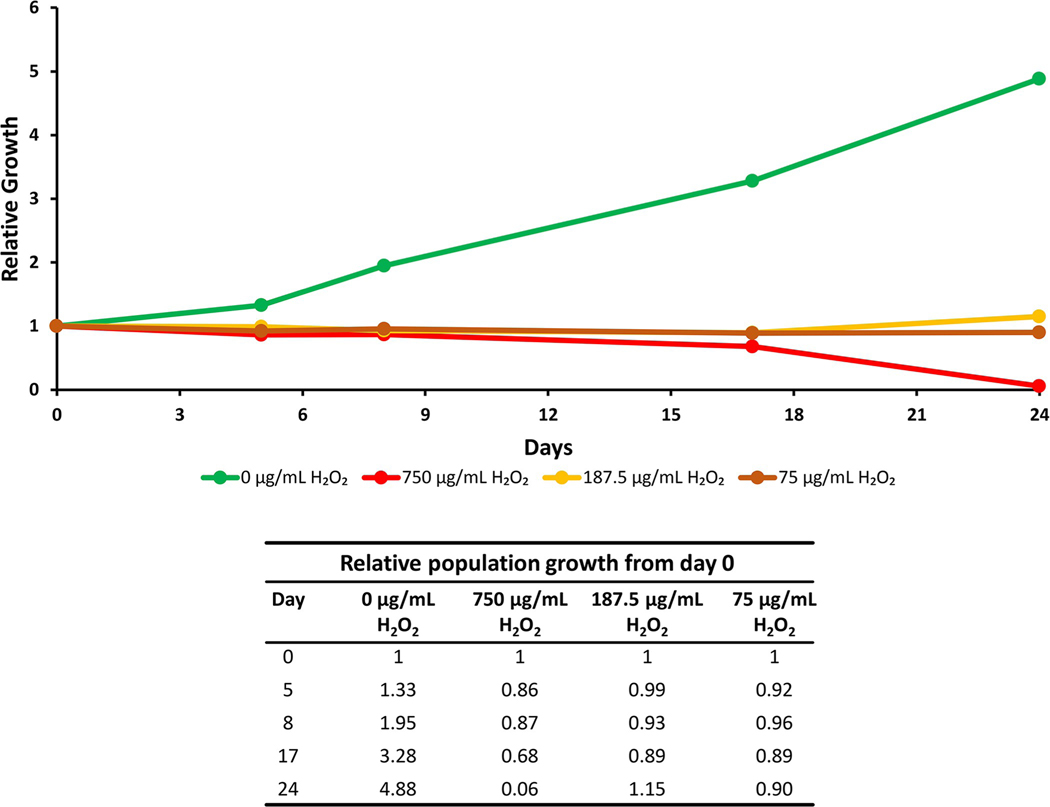
Cyanobacteria growth of treated and control cells. Cyanobacteria growth over 24 days is displayed for control and for 3 doses (75, 187.5, and 750 μg/mL) of H_2_O_2_ treated cells in a line graph and table. The H_2_O_2_ -treated cells did not grow, while the control cells proliferated.

Cyanobacteria cell counts were measured on a flow cytometer from samples treated with different concentrations of H_2_O_2_ (75–750 μg/mL) to compare proliferation, cell death, and changes in fluorescence. It was found that H_2_O_2_ treated samples did not increase in cell count for the 24- hour incubation period, in contrast to the control samples, which continued to proliferate (Fig 11).

**Fig 12.**
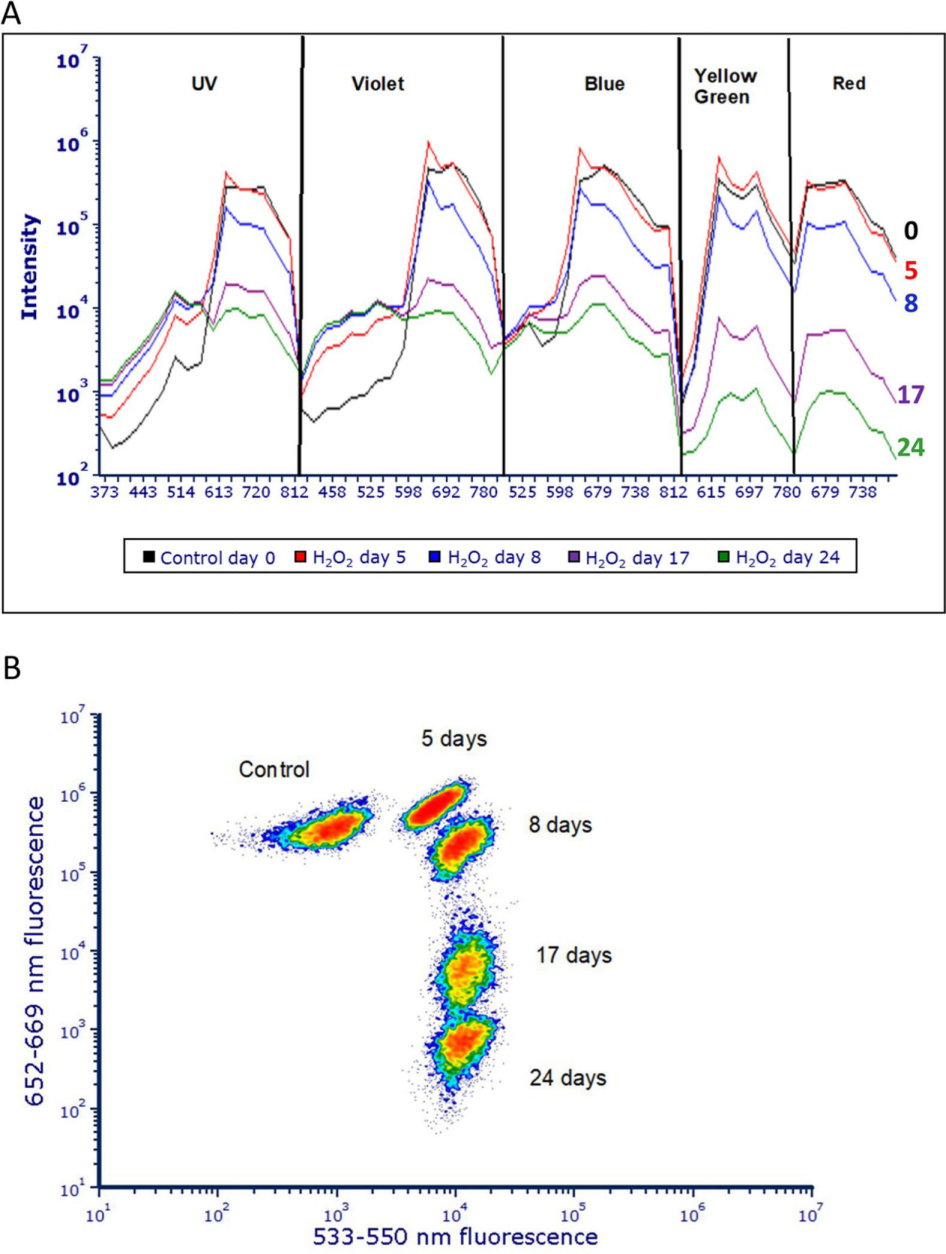
Changes in cyanobacteria fluorescence with 75 μg/mL H_2_O_2_ for 5, 8, 17 and 24 days. The data is shown with fluorescence intensity curves derived from Cytek spectra (A) and cytograms that compare the emission of green fluorescence (533–550 nm) from violet laser excitation and red fluorescence (652–669 nm) from excitation with yellow green laser (B). The changes in the cytogram and spectral maps show a sequential decrease in red fluorescence and an increase in green fluorescence as the H_2_O_2_ interacts with the cells over time.

Control cells didn’t demonstrate a change in fluorescence over the 24 days. The H_2_O_2_ treated cells experienced a sequential decrease in red fluorescence and an increase in green fluorescence, which remained higher than the control for the duration of the experiment. The magnitude of fluorescence intensity changes was dependent on H_2_O_2_ dose and time after the chemical was added. The fluorescence data was derived from the lowest dose (75 μg/mL) and is displayed graphically using the five laser Cytek spectra and cytograms that compare the green fluorescence emissions (533–550 nm) from violet laser excitation and red fluorescence emissions (652–669 nm) from excitation with the yellow-green laser (Fig 12). Changes in red and green fluorescence can be observed even after cyanobacteria growth is inhibited.

## Discussion

The flow cytometer was used to measure the fluorescence response of *M*. *aeruginosa* to different wavelengths of light and concentrations of H_2_O_2_. Spectral FCM has unique properties: the system contains five lasers (355, 405, 488, 561, and 640 nm) with 64 diode detectors, each with a bandpass filter of around 17–25 nm (Cytek Biosciences Inc., Fremont, CA). This provides better spectral detection as the emitted light is acquired with 8–16 bandpass filters with 20–30 nm small detector ranges instead of only 2–5 bandpass detector ranges with a conventional FCM system.

The addition of H_2_O_2_ to *M*. *aeruginosa* produced the following: 1) increased fluorescence intensity in the red (660 nm mean) fluorescence range, 2) increased fluorescence between 428 and 581 nm mostly from the UV, blue and green lasers, and 3) decreased red fluorescence intensity with exposures of 72 hours and longer.

### *M*. *aeruginosa* fluorescence response to H_2_O_2_

*M*. *aeruginosa* fluorescence responded in a dose- and time-dependent manner to H_2_O_2_. Green fluorescence from excitation with the violet laser increased over 3 days with exposure to H_2_O_2_ before declining on day 8 in samples with a dose of 750 μg/mL or higher (Table 2). Red fluorescence derived from yellow-green laser excitation was greater than the control cells in all treated samples until 72 hours of exposure. At 8 days, the red fluorescence of all H_2_O_2_ doses was lower than the control *M*. *aeruginosa*.

The observed increase in red fluorescence 24–48 hours after H_2_O_2_ incubation indicates a release of more photons into the flow cytometer detectors from *M*. *aeruginosa* rather than a utilization of those photons in the production of energy, oxygen, and glucose by the cyanobacteria (Figs 1 and 3). While we did not expect this increase in fluorescence, a few other studies exposing cyanobacteria and microalgae to various treatments, including chemical exposure, high light illumination, extreme heat, and high salinity, have observed an initial increase in fluorescence [6, 14, 22, 26, 27, 33, 34].

Similar to our finding of increased red fluorescence in the 660 nm range, Liu and others showed that strong green light can inhibit energy transfer between PBS pigments in the red alga *Porphyridium cruentum*, resulting in increased emissions between 550–600 nm from PE and decreased emissions in the 660–700 nm range [26]. The authors attributed these effects to photoprotection by preventing the absorption of excessive light, and thus further damage to the cell [26]. Tamary and others also show greater red fluorescence after exposure to high light in *Synechocystis*. They suggest that this occurs due to a decoupling of PBSs from reaction centers which prevents light from entering the photosystems, and thus inhibits photosynthetic electron transport [6]. The result is an increase in 663 nm fluorescence from 580 nm light excitation. This increase in red fluorescence may have occurred due to the detachment or uncoupling of PBSs from the PSII reaction center in response to various stressors, including high-intensity light exposure [5, 6, 26]. In this case, it appears that photons are utilized inefficiently, being absorbed and transmitted through the PBS pigments, but not transferred to PSII and thus emitted as fluorescence to our detectors [6]. Luimstra and others also state that 650–665 nm fluorescence is indicative of decoupled PBSs or PC [8]. We observe a similar effect of increased red fluorescence with FCM. The FCM data shows a fluorescence peak from the treated cells at 660 nm after excitation with the UV, violet, blue, and yellow-green lasers (Figs 4, 6, 8 and 10), which may indicate an inhibition at the PSII reaction center where light is processed. Thus, the 660 nm peak observed in the current study suggests that PBSs may have been decoupled from PSII. The yellow-green laser also shows a peak at 697 nm suggestive of inhibition after absorption of light by chlorophyll.

Backward energy transfer from chlorophyll in the PSII reaction center to the PBS core is one proposed mechanism for PBS decoupling [6]. Backward energy transfer occurs when PSII centers are closed due to a blockage in electron transport between primary electron acceptor Q_A_ and secondary electron acceptor Q_B_ that prevents the oxidation of Q_A_ [12, 35, 36]. The chlorophyll transfers the energy back to the PBSs which then may lead to energetic decoupling of the PBSs [6]. These mechanisms would inhibit light utilization by PSII, resulting in increased fluorescence emissions at wavelengths below 660 nm and a sharp 660 nm peak in the violet, blue, and yellow-green laser channels (Figs 6 and 8).

In the current study, we have shown that H_2_O_2_ exposure results in both an initial increase in red fluorescence as well as decreased red fluorescence at 72 hours and 8 days, an effect potentially linked to photosynthesis inhibition. It is conceivable that PBSs are absorbing photons, but the reaction centers are not fully utilizing them for photosynthesis. *M*. *aeruginosa* samples treated with acetylacetone, DCMU, peracetic acid, and tryptoline exhibited similar fluorescence intensity patterns as those treated with H_2_O_2_, causing a sharp fluorescent peak at 660 nm with UV, violet, blue, and yellow-green laser excitation and increased green fluorescence emissions (Fig 8). Previous research has indicated that acetylacetone, DCMU, and tryptoline cause an inhibition of electron transfer between the primary electron acceptor Q_A_ and the secondary acceptor Q_B_, which blocks PSII [18, 19, 37], while H_2_O_2_ is shown to act on the D1 protein. These results indicate that chemicals with different modes of action may elicit a similar fluorescence response in *M*. *aeruginosa*.

An increase in green fluorescence was observed in *M*. *aeruginosa* samples with almost all H_2_O_2_ doses between 7.5 μg/mL and 3000 μg/mL (Table 2). This increase was not in a specific detector but spread over the whole blue and green spectral ranges detected from UV, violet, and blue laser excitation (Figs 4, 6, 8 and 10). Other studies have found a similar increase in green autofluorescence of perturbed or nonviable cyanobacteria [38–41], but the source of these fluorescence emissions below the 600 nm range remains unclear.

This increase in green fluorescence emissions may be due to upregulation of a green fluorescent molecule in response to H_2_O_2_ exposure. Several studies have investigated autofluorescence in the green spectral range that relates to metabolism and physiology in mammalian cells [42–44] plant cells [45–47], and bacteria [48]. Green and blue fluorescence emissions from mammalian cells is largely attributed to flavins and NADPH [42]. While NADPH and FAD fluorescence have also been studied in cyanobacteria [49, 50], further research is required to determine whether these compounds are contributing to the increased *M*. *aeruginosa* green fluorescence detected from UV, violet, and blue laser excitation recorded in this study.

While the pigments in cyanobacteria absorb yellow-green (561 nm) and red (640 nm) laser light most efficiently, cyanobacteria can also absorb small amounts of lower wavelengths of light from the other three lasers (355 nm, 405 nm, and 488 nm). Due to the short time interval (20 μs) between lasers in the flow cell, pigments excited with one laser may emit fluorescence in another laser path (i.e., emissions from yellow-green laser excitation may be detected in the violet path which is next to the yellow green laser), and this may be the cause the increased red fluorescence intensity and 660 nm peak observed in the UV, violet, and blue laser detectors. Cyanobacteria may be unique in that emission can occur over a longer period of time after the pigments are excited. Generally, the emission from the red laser (80 mW) and yellow green (50 mW) laser are considered very bright as cyanobacteria have pigments that absorb these wavelengths of light. Additionally, cyanobacteria contain photosynthetic pigments and internal structures that result in a different fluorescence and scatter response to bright laser light compared to mammalian cells. It is possible that the biological excitation of the cyanobacteria by one laser may affect the way the cyanobacteria respond to illumination with adjacent lasers. It remains unclear why emissions profiles are similar between UV, violet, and blue laser excitation. Thus, this observation requires further investigation.

If fluorescence emissions from cyanobacteria occur over a second, as has been reported with the bright light illumination of PAM (pulse amplitude modulated) fluorometry and FRRF (fast repetition rate fluorometry), then it is conceivable that this bright light illumination in a flow cytometer may cause pigments to emit light in an adjacent light path to the exciting laser. For example, excitation from the red laser may emit in the UV light path and excitation from the yellow-green laser may emit in the violet and blue light paths as the transit time between light paths is only around 20 μs. However, to examine the possibility of fluorescence spillover, *M*. *aeruginosa* cells or 3 μm Spherotech rainbow beads (excited with all lasers) were run with individual lasers covered to prevent excitation with that wavelength. Covering individual lasers did not appear to significantly affect the fluorescence emissions detected in other laser channels, suggesting that the fluorescence effect observed in this study may not be due to laser emission spillover.

Multiple studies have reported that energy transfer between PBS pigments and chlorophyll in the PSII reaction center occur within picoseconds-nanoseconds [12, 51]. However, PAM fluorometry and FRRF use bright light stimulation which results in a very fast emission of fluorescence followed by a slow (second-long) emission of fluorescence [52, 53]. It is also possible that energy transfer between pigments in cyanobacteria is affected due to the physiological state of the organism [54]. Illumination of cyanobacteria with one laser may affect the way the cells respond to adjacent laser excitation. It remains unclear whether the H_2_O_2_ and light treatments used in this study would cause a delay in fluorescence decay to an extent that fluorescence from one laser is detected in other lasers’ light path detectors or where the H_2_O_2_ would affect the interaction of adjacent lasers with the cyanobacteria resulting in an increase in green fluorescence.

### Synergistic effect of light stress and H_2_O_2_ on *M*. *aeruginosa* fluorescence

In line with previous studies [17, 20, 22, 30, 31], we found a synergistic effect of light and H_2_O_2_ on cyanobacteria fluorescence. The addition of blue and UV-A light to H_2_O_2_ treatments had a greater effect on *M*. *aeruginosa* fluorescence than light or H_2_O_2_ alone, especially at higher light intensities.

Cyanobacteria exhibit photoprotective mechanisms under excessive light exposure by closing their reaction centers and decreasing light utilization [55]. The addition of H_2_O_2_, which damages the D1 protein, inhibits the ability of cells to recover from intense light exposure [17, 20]. For this reason, it appears that the addition of chemicals and light have a synergistic effect on *M*. *aeruginosa*, which is greater than the additive effect of both stimuli. This may be the cause of the synergistic decrease in red fluorescence and increase in green fluorescence observed in response to the UV-A light + H_2_O_2_ treatments (Figs 9 and 10).

Because the fluorescent intensity patterns of the light + H_2_O_2_ samples in Fig 10 were similar to H_2_O_2_ alone (i.e., containing 660 nm peaks), the addition of intense light exposure does not seem to change the mode of damage to the photosynthetic apparatus, but rather intensify the effects of H_2_O_2_ in a non-linear manner.

Our results demonstrate that the effect of light on H_2_O_2_-treated *M*. *aeruginosa* further varies by wavelength. UV-A (395 nm) light had the greatest additive effect on *M*. *aeruginosa* exposed to H_2_O_2_ followed by blue (440–460 nm) light while green light did not affect the cells to the same degree under similar exposure conditions.

UV-A (320–400 nm) light was used in this study as it is more common in the environment compared to UV-B (280–320 nm) and UV-C light (200–280 nm) which are often used in laboratory research studies [56, 57]. While the lower wavelengths UV-B and UV-C are more destructive to mammalian cells and cyanobacteria than UV-A [27, 29], UV-A light is not without its own destructive effects as it has also been shown to cause fluorescence bleaching, photosynthesis, and growth inhibition, D1 protein damage, disruption of electron transfer, and ROS formation [58, 59].

Previous studies have also demonstrated an adverse effect of blue light on cyanobacteria. Luimstra and others reported lower growth rates of *Synechocystis* under blue light compared to orange and red light [8]. Sinha and others suggest that blue light exposure of cyanobacteria caused phycobiliproteins to decouple from PSII. Bleaching of phycobiliproteins under blue light also occurred more quickly than under green light [27]. We hypothesize that this effect under blue light may be due to an imbalance between PSI, which absorbs blue light most efficiently, and PSII, which absorbs green and orange light most efficiently and inhibits photosynthetic electron transport [8, 20, 60–62].

However, Piel and others demonstrated that orange, red, and green light caused a greater decline in photosynthetic vitality of H_2_O_2_-treated *M*. *aeruginosa* cells than blue light. Because PSII absorbs orange light more efficiently than blue light, exposure to orange light is more likely to cause light stress to PSII. This effect combined with damage to the D1 protein and inhibition of PSII repair caused by H_2_O_2_ led to lower photosynthetic vitality [20]. By this logic, in the current study we would expect green light to cause greater changes in red fluorescence relative to the control and blue light, but we did not observe this effect. It is possible that inhibition of photosynthetic electron transport by both blue light illumination [due to a PSI:PSII imbalance; 8] and H_2_O_2_ affected *M*. *aeruginosa* fluorescence more greatly. However, further research should examine the combined effects of blue and green light with H_2_O_2_ on cyanobacteria to better understand this relationship.

### Morphological response (cell size) of *M*. *aeruginosa* to H_2_O_2_ and light stress

FSC has been used by a number of investigators to measure the size of particles as there appears to be a rough correlation between the size of the particle and the amount of forward or small angle scatter [32]. However, there are limitations to using these scatter measurements to quantify the size of particles or organisms like cyanobacteria. There are a number of factors that affect scatter measurements making this parameter both interesting and difficult to study in relation to cellular physiology. The presence of strongly absorbing material like cyanobacteria pigments or textured surfaces will decrease forward scatter. Cells that are dead will have lower refractive indices and thus scatter less light and show a decreased size. The size estimate with cyanobacteria is greatly affected by membrane structures, pigments inside the cell, thylakoids attached to interior membranes, highly textured surfaces, and vacuoles that are used for cellular buoyancy. It was observed that the cyanobacteria responded to H_2_O_2_ by sinking to the bottom of the test tube, demonstrating that H_2_O_2_ affected their buoyancy. This change in vacuoles will greatly affect light scatter signals in different ways dependent on the degree of change within the cells [63, 64]. These parameters decrease the intensity of the FSC signal, resulting in an apparent decrease in cell size. FSC amplitude will not be a monotonic function of particle size, but will be influenced by a number of factors, and thus should only be considered a relative measure.

SSC involves the internal structure of the organism and can be greatly affected by the refractive index and other factors mentioned above for FSC. One of the best examples of this is the difference in SSC of different white blood cells (i.e., granulocytes vs. monocytes/lymphocytes) which varies based on the internal structures contained within the cell [32]. Many of the factors affecting FSC will also affect SSC signals, resulting in measuring a relative size and not an absolute size of the cyanobacteria. The FSC and SSC signals collected in this study reflect changes that are not totally related to the actual physical size of the organism after exposure to H2O2.

In agreement with previous studies [65–67], our results indicate a decrease in relative *M*. *aeruginosa* cell size over time of H_2_O_2_ exposure based on FSC measurements (S1 Table). The addition of high intensity blue and UV-A light to H_2_O_2_ treatments resulted in a general decrease in FSC (S2 Table). It has been suggested that H_2_O_2_ induces oxidative stress and apoptosis [66, 67], and has a similar effect in some mammalian systems [68, 69]. It is clear that H_2_O_2_ caused changes in the cyanobacteria internal structure which may have affected light scatter. Further research is necessary to identify the causes for this decrease in relative size of the organism, but it appears that both membrane alterations and vacuole changes can result in large changes in scatter measurements.

Similar to FSC, SSC also generally decreased with H_2_O_2_ dose and over time (S1 Table). This effect did not appear to be compounded by the light + H_2_O_2_ treatments. Other studies have observed a decrease in SSC in response to H_2_O_2_ exposure, indicating decreased intracellular complexity and granularity [65, 70]. This effect may be due to detachment or degradation of cellular components that contribute to internal complexity, like gas vesicles, [70, 71]. Because H_2_O_2_ targets membranes, it is possible that the decrease in SSC observed in this study is due to damage to the thylakoid membrane.

While most 750 μg/mL H_2_O_2_ + light treatments had lower FSC and SSC than the control, the 750 μg/mL H_2_O_2_ + 7 mW blue light sample exhibited consistently higher FSC values than the control and much lower SSC values compared to other treated samples (S2 Table). This effect may be explained by swelling of cells to a larger volume, which is known to decrease cells’ refractive index and lead to lower SSC measurements [72, 73]. As H_2_O_2_ interacts with the cyanobacteria, the internal structure of the organism will be affected which then affects scatter measurements [63]. However, it is unclear why the H_2_O_2_ + 7 mW blue light sample in particular had highly increased FSC and decreased SSC values relative to the control.

Lastly, light scatter and fluorescence are often positively correlated as larger particles tend to emit higher fluorescence [32]. However, Shapiro warns against using a scatter parameter is not absolute but relative. This makes it potentially laden with variations due to the changing internal parts. We observed higher red fluorescence in the first 48 hours and consistently higher green fluorescent intensity in treated samples that had lower FSC and SSC. The green and red fluorescence intensity were divided by the relative forward light scatter to determine how this indirect size measurement may influence fluorescence. This data suggest that the fluorescent changes in response to H_2_O_2_ were due to physiological or metabolic changes rather than a variation in FSC and SSC measurements.

### Water treatment implications

Despite uncertainty regarding the mechanisms behind these changes, our results demonstrate that examining the fluorescent characteristics of *M*. *aeruginosa* cells may provide information regarding cell physiology and the effects of water treatment chemicals. Fluorescence changes can be observed in this cell culture model system to rapidly identify patterns of cell perturbance, thus serving as an efficient screening tool to determine which treatment chemicals merit deeper investigation for use in managing a given bloom situation.

Our results demonstrate that a flow cytometer can detect fluorescent and morphological differences between control and H_2_O_2_-treated *M*. *aeruginosa* cells in culture. Thus, this machine may be used to assess the efficacy of water treatment chemicals at various doses on a cell culture model. Cyanobacteria samples from lakes cannot usually be run on flow cytometers due to the potential risk of blocking the flow cell. Samples of cyanobacteria from the environment can be measured if the water is filtered through a 37-um nylon mesh filter. However, filtering the water to eliminate large clumps removes a substantial number of organisms and may produce a misrepresentative sample. The flow cytometer can be used to provide foundational data for further research on the efficacy of water treatment chemicals to control HABs in the environment.

Based on red and green fluorescence relative to the control, the three highest H_2_O_2_ doses in this experiment (3000, 1500, 750 μg/mL) were not very different; each exhibited lower red fluorescence than the control after 72 hours of exposure and a decline in green fluorescence at 8 days. Furthermore, by day 8 the ratio of red fluorescence in all treated samples was lower than that of the control, a potential indicator of photosynthesis inhibition. Understanding the effects each dose has on *M*. *aeruginosa* can aid in determining the lowest possible dose to use for treatment as to avoid adverse effects on non-target species.

The results of the current study suggest a synergistic effect of high-intensity light and H_2_O_2_ exposure on *M*. *aeruginosa* which has been reported in other studies [17, 20, 22, 30, 31]. This finding suggests that implementing H_2_O_2_ treatment in water systems may be more effective under exposure to sunlight [30, 31]. Our data also demonstrate that certain wavelengths of light (UV-A and blue) may have a greater effect on *M*. *aeruginosa* than others (green). Some drinking water treatment plants have already utilized UV light to remove other contaminants [74, 75]. Our results suggest that the use of H_2_O_2_ with UV light technology may be an effective method for targeting cyanobacteria in water treatment.

### Potential viability test

The change in green fluorescence from UV, violet, and to a lesser extent blue laser excitation on the flow cytometer can be used to demonstrate cyanobacteria perturbation by different chemical and light conditions. Preliminary data suggest that this increase in green fluorescence is not reversible. Schulze and others [40] found that live *Synechocystis* cells exhibited brighter red fluorescence and nonviable cells emitted green fluorescence using imaging software. The authors suggest that red and green cyanobacteria autofluorescence may be viability indicators. In agreement with Cheloni & Slaveykova, the current study demonstrates that these cyanobacteria autofluorescence parameters can also be detected by FCM [38].

Previous FCM studies on cyanobacteria physiology and viability have measured different parameters (like chlorophyll fluorescence rather than PBS fluorescence) and utilized fluorescent staining [76, 77]. However, we found that *M*. *aeruginosa* autofluorescence was sensitive to chemical and light exposure and may indicate more sensitive and earlier changes in physiology and viability than fluorescent exclusion dyes like SYTOX green, SYTOX orange, SYTO 9, propidium iodide (PI), or DAPI, which only stain membrane-permeable cells. Permeability dyes are a good estimate for dead cells after the membrane has been altered, but these dyes will not enter cells in the initial phase of an interaction with a toxic chemical. We found that PI or SYTOX orange will stain dead cyanobacteria, causing an increase in fluorescence intensity which can be detected on a spectral system. The other permeability dyes that were tried (SYTOX green, DAPI, HO33324, HO33258, and SYTO 9) stained live cells as well as dead cells and were found unsuitable to detect cell death on a flow cytometer under the exposure conditions used. We believe the initial increase in red and green fluorescence occurs before membrane permeability changes allow viability dyes like PI to penetrate into damaged cells. Therefore, this dye permeability assay is not representative of all the dead cells in a population. Additionally, cyanobacteria emit fluorescence in multiple spectral regions, so using fluorescent dyes may obscure the fluorescent patterns recognized in this study and the biophysical factors associated with them. Thus, our findings suggest that it is better not to add a fluorescent dye to show viability change.

The relationship between cyanobacteria growth and changing fluorescence patterns was investigated by measuring cell counts (Fig 11) and spectral fluorescence changes over time (Fig 12). It was found that the samples treated with H_2_O_2_ did not proliferate, while the control samples continued to exhibit increased cell numbers (Fig 11). As the control cells proliferate, their fluorescent characteristics did not change. In contrast, cyanobacteria treated with H_2_O_2,_ showed a sequential decrease in red fluorescence and maintain high green fluorescence over time (Fig 12). H_2_O_2_ treated cyanobacteria cells with decreased red fluorescence and high green fluorescence can be stained with red fluorescing dyes i.e., propidium iodide (PI) or Sytox orange, suggesting they had damaged membranes. These treated cells with low red fluorescence that had permeable membranes were associated with the late stage of cell death. Although the observed early changes consisting of higher red and green fluorescence (Figs 3–6) resulting from H_2_O_2_ treatment cannot be considered a direct assessment of cyanobacteria viability, because the fluorescence change was a consistent toxicological response to H_2_O_2_. It is conceivable that the change in fluorescence may be an early indication of cell death. Further experiments will be necessary to conclusively show these higher fluorescence changes are irreversible and related to viability.

A similar process of increased red fluorescence followed by a decline has been observed with PAM fluorometry and FRRF, which measure fluorescence with a pulse of light after cells have been inoculated in the dark. PAM and FRRF have been used to analyze photosynthetic potential and potential cell damage to algae, cyanobacteria, and plants [52, 53, 61, 78, 79]. H_2_O_2_ and other ROS can cause oxidative damage to cells which can exacerbate photoinhibition likely by targeting PSII repair [80].

A number of researchers have used PAM fluorometry and FRRF to examine fluorescence characteristics of cyanobacteria [14, 20, 22, 25, 52, 60–62, 78, 81, 82]. PAM fluorometry can be used to detect deficiencies in light processing and obtain values like minimal, maximal, and variable fluorescence and approximate other parameters like biomass and photosynthetic vitality [14, 20, 22]). The limitations of this technique, especially in differentiating between PBS and chlorophyll fluorescence, have been described [61, 83, 84]. The purpose of the current study is not to quantify photosynthetic vitality or efficiency but use FCM to detect changes in fluorescence parameters to possibly assess cyanobacteria physiology.

This research may be connected to studies using PAM and FRRF fluorescence equipment. In our study, there was an increase in fluorescence in the red and green ranges when DCMU was added to cyanobacteria (Fig 8), which was similar to the fluorescence observed when H_2_O_2_ was added to the cells for 24–72 hours. This increase in fluorescence observed on the flow cytometer was similar to an observation in a study from Stirbet [52], in which adding DCMU to dark-acclimated cells in the PAM assay showed an increase in fluorescence after excitation with bright light. This FCM technique may be a useful supplement the type of data collected by PAM fluorometry and FRRF to describe cyanobacteria physiology.

## Conclusion

Cyanobacterial blooms are a pervasive environmental issue across the globe. H_2_O_2_ is a common chemical used for treating water systems contaminated with cyanobacteria. Exposure to H_2_O_2_ induces fluorescence changes in *M*. *aeruginosa* which can be detected on a spectral flow cytometer. The use of a spectral flow cytometer helps illuminate the changes between neighboring emission regions due to having smaller bandpass filters to detect fluorescence. Inducing stress with H_2_O_2_ and light exposure revealed greater fluorescent differences between control and treated samples, but also a fluorescent pattern of perturbation in *M*. *aeruginosa* that elucidates the physiological changes that may occur within cyanobacteria after exposure to toxic chemicals. H_2_O_2_ causes an initial fluorescent increase in both the red spectral range (615–829 nm) from yellow-green laser excitation and green spectral range (458–568 nm) from UV, violet, and blue laser excitation, followed by a decrease in red fluorescence and maintained increased green fluorescence. This effect is amplified with the addition of high intensity blue and UV-A light due to a synergistic effect with H_2_O_2_. The 660 nm fluorescence peak in treated samples may occur due to a block in photosynthetic electron flow resulting in PSII inhibition. The cause of increased green fluorescence remains unclear. These fluorescence changes appear to be related to growth inhibition. The green fluorescence emissions potentially indicate damage to *M*. *aeruginosa* which may be an indicator of viability. Despite uncertainty regarding the mechanisms behind these changes, our results demonstrate that examining the fluorescent characteristics of *M*. *aeruginosa* cells can provide information regarding cell physiology and the efficacy of water treatment chemicals. We can use this cell culture model system to rapidly identify patterns of cell perturbance and assess the fluorescent effects of different chemical doses. Thus, FCM can serve as an efficient screening tool to determine which treatment chemicals merit deeper investigation for use in managing a given bloom situation.

## Supplementary Material

SI Table 1 **S1 Table. H**_**2**_**O**_**2**_**-treated *M*. *aeruginosa* forward scatter (FSC) and side scatter (SSC) relative to control.** H_2_O_2_-treated *M*. *aeruginosa* cells exhibited changes in FSC and SSC in a dose- and time-dependent manner. Values indicate ratio of light scatter measurements to the control sample (0 μg/mL H_2_O_2_). FSC and SSC generally decreased over time and with increasing H_2_O_2_ dose. (TIF)

SI Table 3 **S3 Table. Mean red and green fluorescence intensity data from three experiments of cyanobacteria.** The table shows the fluorescence of cells that were treated for 48 hours and 72 hours with 75 μg/mL, and 750 μg/mL of H_2_O_2_. The results (means and SD) of the three individual experiments are displayed.(TIF)

SI Table 2 **S2 Table. Ratio of forward scatter (FSC) and side scatter (SSC) to control in *M*. *aeruginosa* exposed to H**_**2**_**O**_**2**_ **and UV, blue, and green light.** Values indicate ratio of light scatter measurements to the control sample (0 μg/mL H_2_O_2_). Among the light and H_2_O_2_-treated samples, the highest H_2_O_2_ concentration (750 μg/mL) generally exhibited the lowest FSC and SSC values relative to the control. FSC and SSC also generally decreased over time, with a few exceptions. The 7-mW blue + 750 μg/mL sample had unexpected light scatter, with notably higher relative FSC values than the other H_2_O_2_ treatments in that group across all 4 days and extremely low SSC on day 8. The day 8 1 mW UV + 750 μg/mL H_2_O_2_ sample was excluded from analysis due to a low amount of solution remaining that did not allow 20,000 cells to be counted. (TIF)

## Figures and Tables

**Table 1.** *M. aeruginosa* treatment chemicals and doses. Table shows chemicals and doses used to treat *M*. *aeruginosa* culture cells and concentration of chemicals.

Chemical	Formula	Stock concentration (%)	Doses (ug/uL)
Acetylacetone	C_5_H_8_O_2_	2.97	0.74, 0.074, 0.0074
Diuron (DCMU)	C_9_H_10_Cl_2_N_2_O	1.00	0.5, 0.2, 0.1
Hydrogen peroxide	H_2_O_2_	3.00	3, 1.5, 0.75, 0.3, 0.15, 0.075, 0.03, 0.015, 0.0075, 0.003
Peracetic acid	C_2_H_4_O_3_	3.20	0.8, 0.08, 0.008, 0.0008
Tryptoline	C11H12N2	1.00	0.075, 0.0075, 0.00075

**Table 2.** Red and green fluorescence of *M*. *aeruginosa* cells exposed to H_2_O_2_ over time relative to control. H_2_O_2_-treated *M*. *aeruginosa* cells exhibited changes in red (emission: 652–669 nm, excitation: 561 nm) and green (emission: 533–550 nm, excitation: 405 nm) fluorescence in a dose- and time-dependent manner as shown in Fig 1. Values indicate ratio of fluorescence to the control sample (0 μg/mL H_2_O_2_). Treated cells initially emitted increased red fluorescence (24–48 hours) followed by a decline starting at 72 hours for high dose samples (300–3000 μg/mL) and 8 days for the remaining treated samples. Green fluorescence increased in most treated samples for the first three days before declining in high dose samples after 8 days. The green fluorescence of all treated samples remained higher than the control. Numbers highlighted red in the chart indicate at least 30% lower fluorescence than control. Ratios of 0.00 indicate numbers too small to show with two decimal places.

	24 hours		48 hours		72 hours		8 days	
Dose (ug/uL)	Red ratio to control	Green ratio to control	Red ratio to control	Green ratio to control	Red ratio to control	Green ratio to control	Red ratio to control	Green ratio to control
**0**	1	1	1	1	1	1	1	1
**0.003**	0.95	1.05	0.95	1.02	0.91	1.00	0.96	1.03
**0.0075**	1.22	5.67	1.44	5.34	1.26	6.34	0.23	11.52
**0.015**	1.93	8.37	2.50	9.04	1.81	10.35	0.08	16.81
**0.03**	1.98	6.60	2.83	7.06	2.14	9.15	0.07	15.72
**0.075**	2.26	5.19	3.31	5.52	2.39	8.39	0.03	12.80
**0.15**	2.56	5.27	3.27	5.76	2.29	8.13	0.02	10.32
**0.3**	3.06	4.74	3.03	6.33	1.56	8.81	0.00	7.53
**0.75**	3.39	5.03	2.56	7.44	0.88	9.92	0.00	5.24
**1.5**	3.28	5.54	2.07	8.34	0.60	10.42	0.00	4.59
**3**	2.75	6.73	1.36	10.25	0.25	11.15	0.00	4.70

**Table 3.** Average green and red fluorescence for three separate experiments at 48 and 72 hours after H_2_O_2_ exposure. The data for each individual experiment are shown in S3 Table.

Average fluorescence intensities ± SD		
48 hours		
Dose (μg/mL)	Green Fl	Red Fl
0	1,813 ± 124	393,106 ± 60,112
75	8,071 ± 503	775,823 ± 184,831
750	10,987 ± 1,455	969,993 ± 165,199
72 hours		
Dose (μg/mL)	Green Fl	Red Fl
0	1,935 ± 88	395,512 ± 261,275
75	9,778 ± 1,521	904,363 ± 261,275
750	14,964 ± 2,229	748,050 ± 179,621

**Table 4.** Red and green fluorescence of *M*. *aeruginosa* cells exposed to H_2_O_2_ and UV, blue, and green light over time relative to control. Cells were exposed to 3 different concentrations of H_2_O_2_ and different illumination conditions with UV-A (0.5-mW and 1-mW), blue light (7-mW and 14-mW), and green light (6-mW and 15-mW). These data indicate a synergistic effect of H_2_O_2_ + UV-A light (395 nm) and blue light (440–460 nm) dependent on H_2_O_2_ dose and exposure time, and light intensity, which correlate with the data shown in Fig 7 for the 24- and 72-hour time points. Red fluorescence refers to 652–669 nm fluorescence with 561 nm excitation, and green fluorescence refers to 533–550 nm fluorescence with 405 nm excitation. Numbers highlighted red indicate at least 30% lower fluorescence than control. Ratios of 0.00 indicate numbers too small to show with two significant figures.

	Light exposure 2 (24 hours)		48 hours		72 hours		8 days	
H_2_O_2_ dose (ug/uL)	Red ratio to control	Green ratio to control	Red ratio to control	Green ratio to control	Red ratio to control	Green ratio to control	Red ratio to control	Green ratio to control
**No light**								
**control**	1.00	1.00	1.00	1.00	1.00	1.00	1.00	1.00
**0.0075**	1.00	1.02	1.00	0.91	0.98	0.91	0.90	1.03
**0.075**	1.56	4.40	1.71	4.14	1.95	5.13	0.99	12.89
**0.75**	2.61	5.09	2.52	6.08	1.75	8.24	0.01	8.33
**0.5 mW UV**								
**control**	0.97	0.98	0.96	0.88	0.95	0.87	0.94	1.00
**0.0075**	0.99	0.96	0.90	0.86	0.87	1.15	0.66	3.24
**0.075**	1.65	3.73	1.86	3.57	2.23	4.94	0.69	14.76
**0.75**	2.62	5.16	2.38	6.30	1.79	8.27	0.02	9.20
**1 mW UV**								
**control**	0.79	0.94	0.71	0.89	0.72	0.89	0.82	1.05
**0.0075**	1.48	11.83	1.30	13.12	1.15	15.23	0.49	20.61
**0.075**	0.72	16.30	0.43	17.29	0.30	18.00	0.04	20.00
**0.75**	0.51	12.57	0.16	12.79	0.05	11.87	N/A	N/A
**7 mW blue**								
**control**	0.98	0.98	1.01	0.84	0.96	0.84	0.91	0.97
**0.0075**	1.00	0.99	1.00	0.89	0.99	0.88	0.92	1.02
**0.075**	1.77	5.02	1.88	3.82	2.19	4.92	0.86	13.32
**0.75**	3.09	5.20	2.77	6.49	1.72	7.72	0.00	9.26
**14 mW blue**								
**control**	1.06	7.32	1.04	2.30	0.99	1.85	0.93	3.88
**0.0075**	1.93	11.26	1.63	12.96	1.44	14.92	0.66	19.49
**0.075**	2.12	10.06	1.71	12.93	1.42	15.29	0.46	20.03
**0.75**	2.06	9.63	1.46	13.17	0.89	14.44	0.00	11.15
**6 mW green**								
**control**	0.99	0.98	1.06	0.92	1.01	0.88	0.89	1.00
**0.0075**	1.03	2.59	1.10	1.55	1.14	1.68	0.71	7.77
**0.075**	1.56	4.91	1.62	4.11	1.84	4.79	1.31	10.88
**0.75**	2.69	5.22	2.54	5.81	1.86	7.72	0.01	9.41
**15 mW green**								
**control**	0.98	1.01	0.96	0.87	0.94	0.83	0.98	0.98
**0.0075**	1.00	2.10	0.97	1.09	0.96	0.97	0.63	1.89
**0.075**	1.61	4.96	1.78	4.14	2.06	5.10	1.01	12.50
**0.75**	2.59	6.61	2.15	7.14	1.53	8.91	0.02	9.88

## Data Availability

The data that was obtained is the sole property of the EPA and is not owned by any commercial company. We use FCS express software (third-party) as it has added features that can be used to analyze Cytek spectral data that are not available in the Cytek Spectra Flo operating system. The data belongs to the EPA and not the Cytek company or a third-party company. The data is collected on a Cytek Aurora flow cytometer which is owned by the EPA. The standard procedure that we use with the generated data is to convert the Spectra Flo data into FCS 3.0 files which can be used by an EPA scientist or by other investigators that have third-party software. Converting data from a flow cytometer to FCS files is a standard procedure used by the flow cytometry community to analyze data for further analysis. The authors did not have any special access privileges from the Cytek company or a third-party software company. The Spectra Flo data can be used by individuals that have a Cytek flow cytometer or have access to any third-party software that can read FCS 3.0 files.
