# Supplementary figures and images for: Analysis of Microcystis aeruginosa physiology by spectral flow cytometry: Impact of chemical and light exposure
Source: PLOS Water. Author manuscript; Available in PMC 2024 Oct 27. (PMC10953801; doi:10.1371/journal.pwat.0000177)

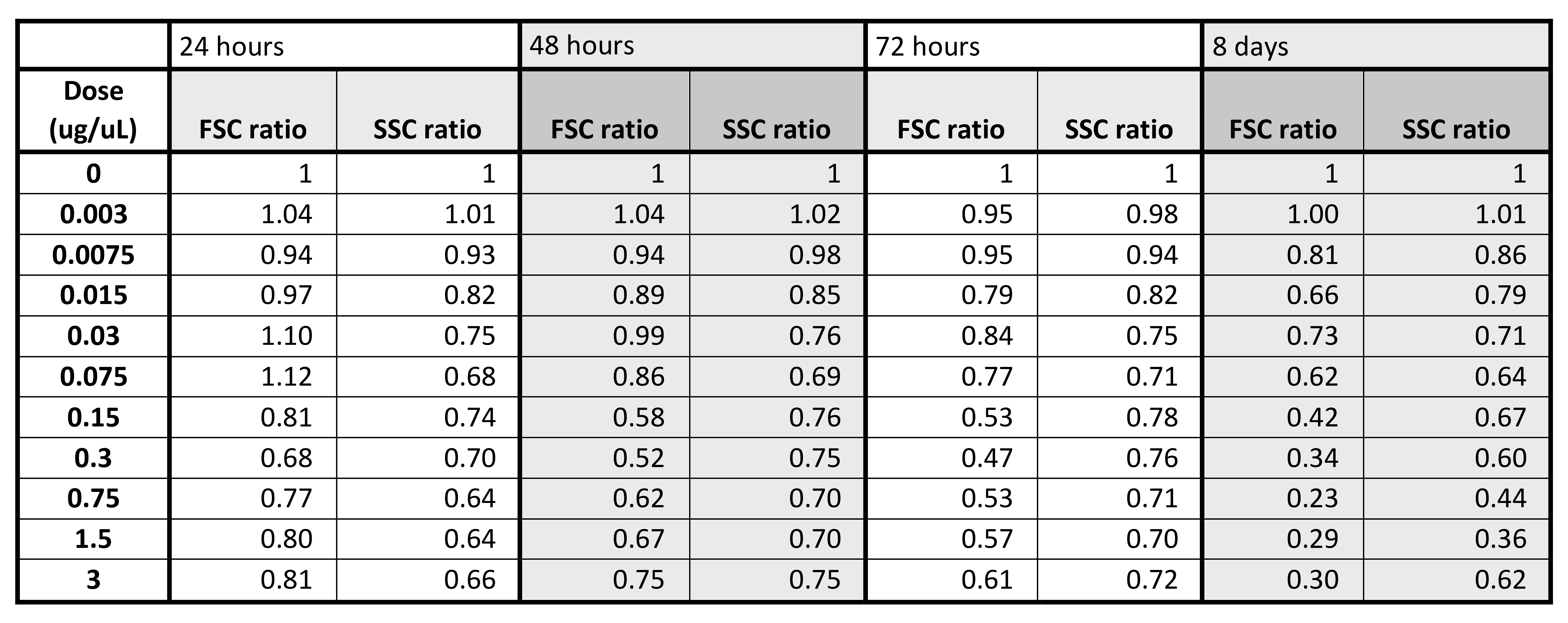

Supplement: SI Table 1 — S1 Table. H2O2-treated M. aeruginosa forward scatter (FSC) and side scatter (SSC) relative to control. H2O2-treated M. aeruginosa cells exhibited changes in FSC and SSC in a dose- and time-dependent manner. Values indicate ratio of light scatter measurements to the control sample (0 μg/mL H2O2). FSC and SSC generally decreased over time and with increasing H2O2 dose. (TIF) [file NIHMS1947148-supplement-SI_Table_1.tif]

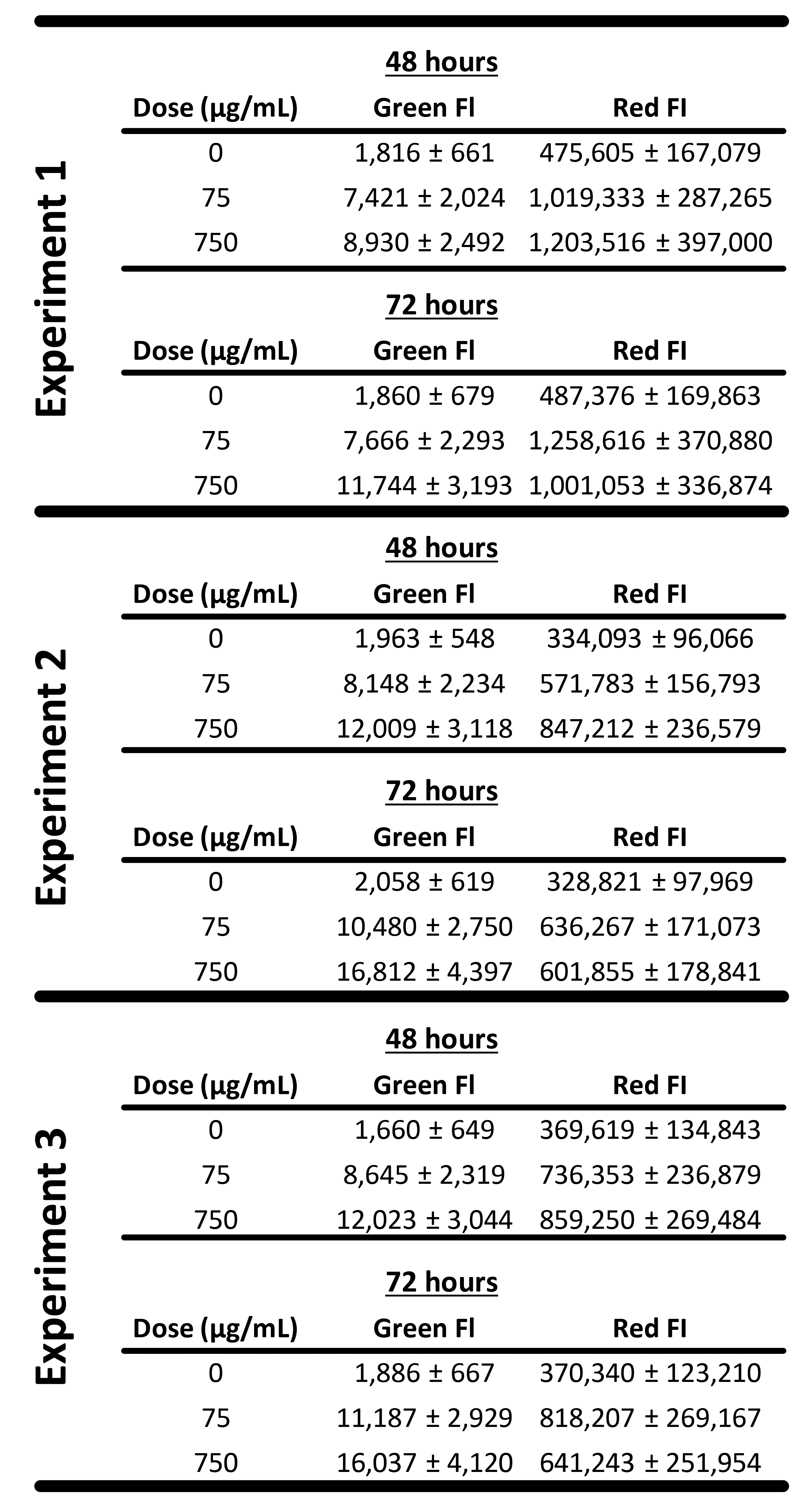

Supplement: SI Table 3 — S3 Table. Mean red and green fluorescence intensity data from three experiments of cyanobacteria. The table shows the fluorescence of cells that were treated for 48 hours and 72 hours with 75 μg/mL, and 750 μg/mL of H2O2. The results (means and SD) of the three individual experiments are displayed.(TIF) [file NIHMS1947148-supplement-SI_Table_3.tif]
